# Phenalenyl-based mononuclear dysprosium complexes

**DOI:** 10.3762/bjnano.7.92

**Published:** 2016-07-08

**Authors:** Yanhua Lan, Andrea Magri, Olaf Fuhr, Mario Ruben

**Affiliations:** 1 Institut für Nanotechnologie, Karlsruher Institut für Technologie (KIT), Postfach 3640, D-76344 Karlsruhe, Germany (Tel: +49 721-608-28948); 2 Institut Néel, CNRS, Nanosciences Department, BP 166, 25 rue des Martyrs, 38042 GRENOBLE Cedex 9, France; 3 Karlsruhe Nano Micro Facility (KNMF), Karlsruher Institut für Technologie (KIT), Postfach 3640, D-76344 Karlsruhe, Germany; 4 Université de Strasbourg, Institut de Physique et de Chimie des Materiaux de Strasbourg, Campus de Cronenbourg, 23 Rue du Loess, 67034 Strasbourg Cedex 2, France

**Keywords:** coordination complexes, dysprosium, magnetism, mononuclear, phenalenyl-based

## Abstract

The phenalenyl-based dysprosium complexes [Dy(PLN)_2_(HPLN)Cl(EtOH)] (**1**), [Dy(PLN)_3_(HPLN)]·[Dy(PLN)_3_(EtOH)]·2EtOH (**2**) and [Dy(PLN)_3_(H_2_O)_2_]·H_2_O (**3**), HPLN being 9-hydroxy-1*H*-phenalen-1-one, have been synthesized. All compounds were fully characterized by means of single crystal X-ray analysis, paramagnetic ^1^H NMR, MALDI-TOF mass spectrometry, UV–vis spectrophotometry and magnetic measurements. Both static (dc) and dynamic (ac) magnetic properties of these complexes have been investigated, showing slow relaxation of magnetization, indicative of single molecule magnet (SMM) behavior. Attempts to synthesize sublimable phenalenyl-based dysprosium complexes have been made by implementing a synthetic strategy under anhydrous conditions. The sublimed species were characterized and their thermal stability was confirmed. This opens up the possibility to deposit phenalenyl-based lanthanides complexes by sublimation onto surfaces, an important prerequisite for ongoing studies in molecular spintronics.

## Introduction

In the pioneering studies of next-generation information processing devices, single-molecule magnets (SMMs) are often regarded as building blocks for realizing spintronic devices at the molecular scale [1]. Out of thousands of SMMs available, the class of the LnPc_2_ (Ln = Tb, Dy) complexes is the most popular example. It has been successfully assembled into different molecular schemes of spintronic quantum devices such as spin valve, spin transistor and spin resonator [2–5]. The fascinating experimental results obtained from these quantum devices foster a bright future in the field of nanospintronics.

In order to fabricate spintronic devices with molecular units, an essential step is to investigate the magnetic properties of the molecules on the surface of or close to conducting electrodes. So far, depositing and addressing individual molecules of SMMs on surfaces [2] was explored only for very few complexes such as TbPc_2_, Fe_4_ and Mn_12_. With the successful experience in utilizing TbPc_2_ [6], our earnest attention in searching new SMMs with enhanced properties has led to the preparation of mononuclear lanthanide complexes. Indeed the mononuclear lanthanide complexes could allow for the study of controlled entanglement of spins on neighboring spin carriers [7], because there is no decoherence of individual spins through dipolar exchange in such a simple molecular unit. As highlighted recently in many work [8–14], the determining factor in the construction of SMMs and single-ion magnets (SIMs) and in the control of the single-axial anisotropy is related to the symmetry at the individual Ln sites along with the nature of the ligand field. Owing to a very strong Ising-type uniaxial anisotropy of the metal ion, dysprosium containing compounds are extensively studied and have been found to have the most promising slow-relaxation behavior. Typical examples are [Dy(acac)_3_(H_2_O)_2_] [8] and known organometallic compounds such as [Li(THF)_4_][(Ph_2_PNPh)_4_Dy] and [Dy{N(PPh_2_)_2_}_3_] [15], Dy-(N(SiMe_3_))_3_ [16] and [{Cp’_2_Dy(µ-SSiPh_3_)}_2_] (Cp’= η^5^-C_5_H_4_Me) [17], [Dy(1,4-(Me_3_Si)_2_-C_8_H_6_)_2_] [18], and [Dy(COT’’)_2_Li(THF)(DME)] (COT’’ = 1,4-bis(trimethylsilyl)cyclooctatetraenyl dianion) [19] showing interesting magnetic relaxation properties.

A phenalenyl unit can be considered as an aromatic variation of β-diketonates (Figure S1, Supporting Information File 1) formed by the fusion of three benzene rings. As a consequence of the extended aromatic system, enhanced interactions with carbon-based surfaces such as HOPG, graphene and CNT substrates are expected [20]. The significance of the phenalenyl unit in the diverse research areas ranging from chemistry and materials chemistry to device physics is closely linked to its essential role as an electronic reservoir that has driven the development of the best organic single-component conductor and has led to the creation of spin memory devices [21]. Indeed, a neutral planar phenalenyl-based molecule namely zinc methyl phenalenyl has recently been utilized as non-innocent building block for the construction of a spin memory device by Raman and co-workers [22]. Upon deposition of these molecules onto a ferromagnetic surface, a hybridized organometallic supramolecular magnetic layer can be formed. This interface layer exhibits a spin-dependent resistance leading to an interface magnetoresistance (IMR) effect. These findings suggest that phenalenyl-based molecules could potentially be utilized in building molecular-scale quantum spin memory and processors.

Taking all these points into consideration, one idea would be to step further and to introduce anisotropic lanthanide ions into these phenalenyl-based complexes with the focus on their interesting magnetic properties. In this regard, the 9-hydroxy-1*H*-phenalen-1-one (HPLN) ligand has previously been utilized for the preparation of mononuclear lanthanide complexes [23–24] to study the near-infrared luminescence upon excitation with visible light. However, this work, carried out by our group, aimed to study gas-phase dispersed photoluminescence spectra [25–26]. In this paper we report on the synthesis of three mononuclear dysprosium complexes [Dy(PLN)_2_(HPLN)Cl(EtOH)] (**1**), [Dy(PLN)_3_(HPLN)]·[Dy(PLN)_3_(EtOH)]·2EtOH (**2**) and [Dy(PLN)_3_(H_2_O)_2_]·H_2_O (**3**) with the focus on studying their magnetic properties. In addition, these complexes have been characterized by means of single crystal X-ray analysis, paramagnetic ^1^H NMR, MALDI–TOF spectrometry and UV–vis spectrophotometry. Furthermore, attempts to synthesize sublimable phenalenyl-based dysprosium complexes **4** have been made. The sublimed species **4’** was characterized confirming its thermal stability.

## Results and Discussion

### Synthesis

**Scheme 1:**
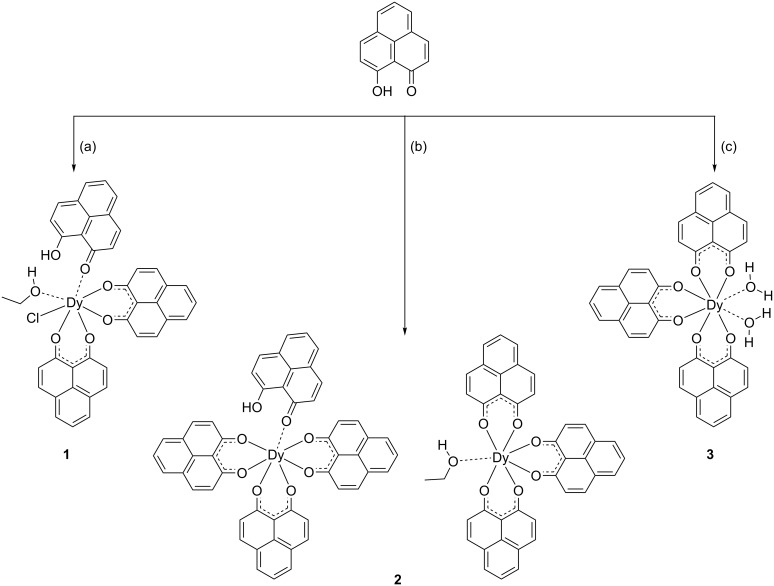
Synthesis of complexes **1**–**3**. (a) NaH, 1/3DyCl_3_·6H_2_O, EtOH, reflux then stirring at RT overnight. (b) Diisopropylamine, 1/3DyCl_3_·6H_2_O, EtOH, reflux then stirring at RT overnight. (c) Diisopropylamine, 1/3DyCl_3_·6H_2_O, big volume of EtOH, reflux then stirring at RT overnight.

Van Deun et al. [23–24] synthesized 1:3 metal-to-ligand complexes using ammonia as a base and 1:4 metal-to-ligand complexes using the stronger base NaOH. In addition, phenalenyl-based europium and gadolinium nonanuclear complexes [26] were obtained by using (Et)_3_N as base in a 1:1.8 metal-to-ligand ratio. The synthetic procedures reported in our work are illustrated in Scheme 1. Instead of ammonia or NaOH as a base [23–24], we used NaH or diisopropyl amine to deprotonate the HPLN ligand. In order to obtain neutral complexes and to make them sublimable, we carried out the reactions only in a 1:3 Dy/HPLN stoichiometry. In general, the reaction is maintained at room temperature and then refluxed for 3 h. The crystals are grown through slow evaporation from the filtrate. Complex [Dy(PLN)_2_(HPLN)Cl(EtOH)] (**1**) was formed in a mixed solvent of CHCl_3_/EtOH (1:5) in the presence of NaH, while complex [Dy(PLN)_3_(HPLN)]·[Dy(PLN)_3_(EtOH)]·2EtOH (**2**) was obtained in pure EtOH using diisopropyl amine as a base. Since two mononuclear species are co-crystallized in **2**, the volume of EtOH is then scaled up to the 1.5-fold, resulting in [Dy(PLN)_3_(H_2_O)_2_]·H_2_O (**3**).

**Scheme 2:**
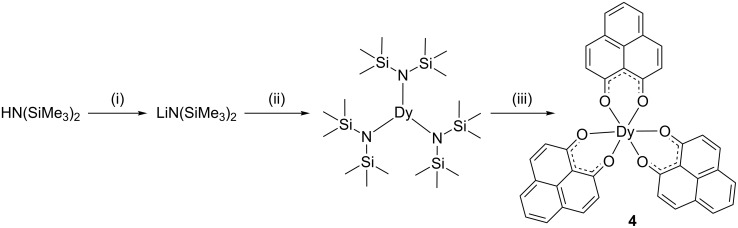
Synthesis of complex **4** under anhydrous conditions. The proposed structure of the complex is based on the characterizations. (i) *n*-BuLi, THF, 0 °C, 1 h. (ii) 1/3DyCl_3_, THF, stirring at RT overnight. (iii) 3HPLN, THF, reflux overnight.

Due to the presence of non-depronated ligands and solvent molecules in the coordination sphere, all these complexes decomposed at ca. 350 °C during the sublimation process in high vacuum (10^−6^ mbar). Sublimable lanthanides quinolinates have been prepared by Katkova et al. by using bis(trimethylsilyl)amino complexes as precursor [27–29]. With this experience, a sublimable phenalenyl-based dysprosium complex **4** was synthesized as illustrated in Scheme 2. The synthesis was carried out under anhydrous conditions implementing standard Schlenk techniques, carefully distilled THF, anhydrous starting materials and oven-dried glassware. So far, single crystals of the complex **4** were not obtained, thus its exact structure remains unknown yet. Nevertheless, the product was fully characterized by NMR spectroscopy, mass spectrometry and UV–vis spectrophotometry in comparison to the complexes **1**–**3**. In addition, complex **4** was successfully sublimated at about 300 °C in high vacuum (10^−6^ mbar). To confirm the thermal stability, the compound was also characterized after the sublimation process.

### X-ray crystal structures

**Figure 1:**
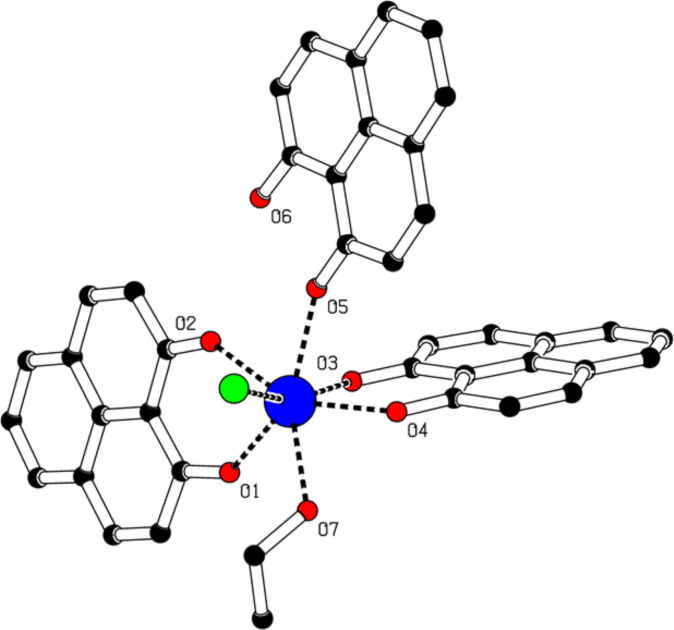
Molecular structure of complex **1** obtained by single crystal X-ray diffraction. Hydrogen atoms are omitted for clarity. Selected bond lengths [Å], angles [°]: Dy-Cl1 2.6983(5), Dy-O1 2.2517(18), Dy-O2 2.2647(14), Dy-O3 2.2802(15), Dy-O4 2.2599(16), Dy-O5 2.3756(16), Dy-O7 2.4215(17); Cl-Dy-O1 108.38(4), O2-Dy-O3 85.95(5), Cl-Dy-O2 92.92(4), O2-Dy-O4 149.03(6), Cl-Dy-O3 161.85(4), O2-Dy-O5 72.01(6), Cl-Dy-O4 99.30(4), O2-Dy-O7 139.73(6), Cl-Dy-O5 84.30(4), O3-Dy-O4 73.69(5), Cl-Dy-O7 79.19(4), O3-Dy-O5 78.12(5), O1-Dy-O2 73.31(5), O3-Dy-O7 112.98(5), O1-Dy-O3 88.68(6), O4-Dy-O5 80.97(6), O1-Dy-O4 127.84(5), O4-Dy-O7 70.92(6), O1-Dy-O5 143.56(5), O5-Dy-O7 144.33(6), O1-Dy-O7 72.06(6).

**Figure 2:**
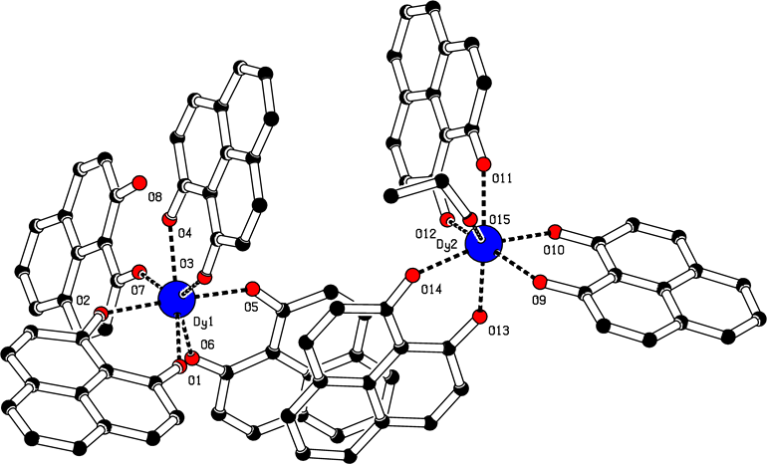
Molecular structure of complex **2** obtained by single crystal X-ray diffraction. Hydrogen atoms are omitted for clarity. Selected bond lengths [Å], angles [°] for **2a**: Dy1-O1 2.290(3), Dy1-O2 2.286(3), Dy1-O3 2.280(3), Dy1-O4 2.280(3), Dy1-O5 2.289(3), Dy1-O6 2.309(3), Dy1-O7 2.381(3); O1-Dy1-O2 73.25(11), O3-Dy1-O4 72.93(12), O1-Dy1-O3 73.37(12), O3-Dy1-O5 86.47(12), O1-Dy1-O4 142.63(12), O3-Dy1-O6 137.28(11), O1-Dy1-O5 105.15(11), O3-Dy1-O7 144.34(12), O1-Dy1-O6 77.00(12), O4-Dy1-O5 88.42(11), O1-Dy1-O7 138.78(11), O4-Dy1-O6 140.20(12), O2-Dy1-O3 95.61(12), O4-Dy1-O7 71.55(12), O2-Dy1-O4 94.47(11), O5-Dy1-O6 72.33(11), O2-Dy1-O5 176.83(12), O5-Dy1-O7 95.51(11), O2-Dy1-O6 104.56(11), O6-Dy1-O7 75.94(12), O2-Dy1-O7 84.20(11). Selected bond lengths [Å], angles [°] for **2b**: Dy2-O9 2.324(3), Dy2-O10 2.268(3), Dy2-O11 2.249(3), Dy2-O12 2.288(3), Dy2-O13 2.290(3), Dy2-O14 2.261(3), Dy2-O15 2.444(3); O9-Dy2-O10 72.83(12), O11-Dy2-O12 73.47(12), O9-Dy2-O11 117.23(11), O11-Dy2-O13 154.72(12),O9-Dy2-O12 156.73(12), O11-Dy2-O14 112.83(12), O9-Dy2-O13 82.10(11), O11-Dy2-O15 72.58(13), O9-Dy2-O14 107.08(12), O12-Dy2-O13 82.82(12), O9-Dy2-O15 73.15(12), O12-Dy2-O14 85.15(12),O10-Dy2-O11 82.04(12), O12-Dy2-O15 129.87(12), O10-Dy2-O12 89.34(12), O13-Dy2-O14 72.88(12), O10-Dy2-O13 89.16(11), O13-Dy2-O15 131.41(12), O10-Dy2-O14 161.72(12), O14-Dy2-O15 75.44(12), O10-Dy2-O15 120.73(12).

**Figure 3:**
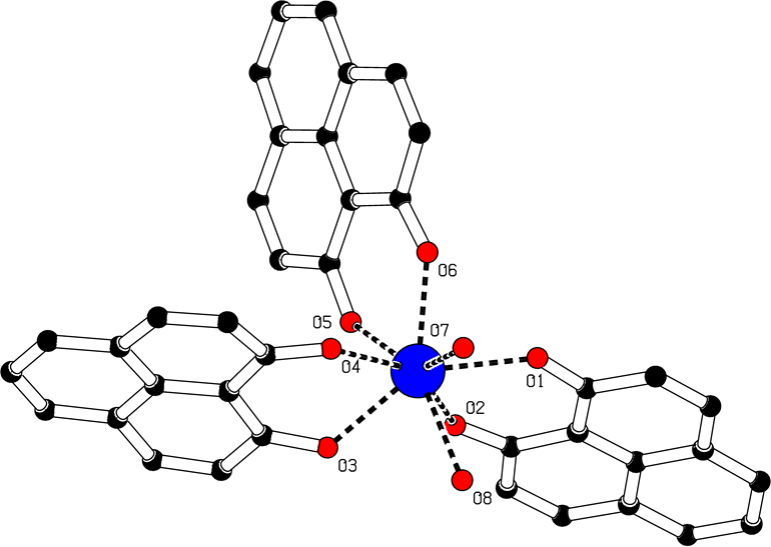
Molecular structure of complex **3** obtained by single crystal X-ray diffraction. Hydrogen atoms are omitted for clarity. Selected bond lengths [Å], angles [°]: Dy1-O1 2.347(3), Dy1-O2 2.332(4), Dy1-O3 2.337(3), Dy1-O4 2.339(4), Dy1-O5 2.317(4), Dy1-O6 2.323(3), Dy1-O7 2.396(3), Dy1-O8 2.439(4); O1-Dy-O2 70.56(12), O3-Dy-O5 74.38(12), O1-Dy-O3 143.35(12), O3-Dy-O6 134.79(13), O1-Dy-O4 139.12(11), O3-Dy-O7 122.26(11), O1-Dy-O5 116.79(12), O3-Dy-O8 75.98(12), O1-Dy-O6 79.60(12), O4-Dy-O5 89.53(13), O1-Dy-O7 72.29(11), O4-Dy-O6 80.61(12), O1-Dy-O8 76.93(12), O4-Dy-O7 68.10(12), O2-Dy-O3 79.57(12), O4-Dy-O8 101.23(13), O2-Dy-O4 149.73(12), O5-Dy-O6 71.05(12), O2-Dy-O5 76.36(13), O5-Dy-O7 141.52(12), O2-Dy-O6 118.31(12), O5-Dy-O8 142.94(11), O2-Dy-O7 136.96(13), O6-Dy-O7 74.55(11), O2-Dy-O8 76.84(13), O6-Dy-O8 145.39(12), O3-Dy-O4 70.80(11), O7-Dy-O8 74.29(11).

Single crystals of the complexes **1**–**3** were grown through the slow evaporation from the respective reaction filtrate and their structures were determined by single crystal X-ray diffraction. The refinement data are summarized in Table S1 (Supporting Information File 1) and the bond lengths and angles are listed in the caption of Figures **1**–**3**. The molecular structure of [Dy(PLN)_2_(HPLN)Cl(EtOH)] (**1**) is illustrated in Figure 1. The central dysprosium atom is coordinated by seven donor atoms, six oxygen and one chlorine, resulting in a mono-capped trigonal prism geometry with a symmetry lower than *D*_5_*_h_*. The bond lengths Dy-O1, Dy-O2, Dy-O3 and Dy-O4 (of the deprotonated phenalenyl) are between 2.25 and 2.28 Å. In addition, the bond angles Dy-O1-C1, Dy-O2-C3, Dy-O3-C14, and Dy-O4-C16, range between 136.8 and 138.8°. The bond length of Dy-Cl is 2.70 Å, which is longer than the Dy-O bod lengths. Although the molecule itself is asymmetric, the two deprotonated phenalenyls are virtually identical. On the contrary, compared to those of the deprotonated phenalenyls, the bond length Dy-O5 of the protonated phenalenyl of 2.38 Å is considerably longer and the angle Dy-O5-C27 of 148.3° is wider. There are two intramolecular hydrogen bonds between O6H6 and O2 (2.52 Å) and between O6H6 and O5 (1.90 Å) associated with the non-depronated phenalenyl, and an intermolecular hydrogen bond between O7H7 and Cl1 (2.49 Å) involving EtOH. Clearly, the intermolecular hydrogen bonds, together with the π–π stacking (ca. 3.45 Å) between the phenalenyl moieties, aid the molecular packing in the crystal lattice (Figure S2, Supporting Information File 1).

In an asymmetric unit of complex **2**, two mononuclear dysprosium moieties [Dy(PLN)_3_(HPLN)] **2a** and [Dy(PLN)_3_(EtOH)] **2b** are co-crystallized together with two EtOH lattice molecules as illustrated in Figure 2. In both moieties the central dysprosium is coordinated by seven oxygen atoms in a mono-capped trigonal prism geometry. Compared with those of the protonated and deprotonated phenalenyls, the bond lengths and angles are down to the same trend as that described for complex **1**, in which those for the deprotonated phenalenyls are longer and wider than those of the protonated ones. On the other hand, compared to those around each dysprosium atom in the two moieties of **2a** and **2b**, the bond lengths and angles are very similar. The bond lengths between the dysprosium and the oxygen atoms of the protonated phenalenyls vary from 2.280 to 2.309 Å for **2a** and 2.249 to 2.324 Å for **2b**; and the bond angles range from 135.3 to 139.1° for **2a** and from 137.5 to 139.1° for **2b**. It is noteworthy that the bond distances between the dysprosium atom and the oxygen atoms of the HPLN ligands can be compared to those reported in other dysprosium–oxygen compounds [30–36]. Additionally, two intramolecular hydrogen bonds are formed: O8H8-O4 (2.39 Å) and O8H8-O7 (1.86 Å) in **2a**, but three are formed in **2b**. The intermolecular hydrogen bond, O15H15-O16 (2.06 Å) involves the EtOH bonded to the dysprosium and one EtOH in the crystal lattice. Two more hydrogen bonds, O16H16-O9 (2.06 Å) and O17aH17a-O12 (2.23 Å), take place between the deprotonated phenalenyls and one EtOH in the crystal lattice. As clearly depicted in Figure S3 (Supporting Information File 1), the presence of EtOH molecules in the crystal lattice forming intermolecular hydrogen bonds packs the molecules together and drives the co-crystallization. Additionally, weak π–π stacking (ca. 3.61 Å) between the phenalenyl moieties of **2a** and **2b** might be also responsible for the co-crystallization of the complexes in one asymmetric unit.

Compound [Dy(PLN)_3_(H_2_O)_2_]·H_2_O (**3**) crystallizes in a monoclinic system in contrast to the triclinic one of **1** and **2**. The central dysprosium is surrounded by three anionic phenalenyls and two water molecules (Figure 3), in which the eight chelating oxygen atoms result in a distorted square antiprismatic arrangement. The bond lengths between the dysprosium and the oxygens of the deprotonated phenalenyls are in the range of 2.317 and 2.347 Å. These distances are relatively longer than those observed in **1**, **2a** and **2b**, in which the dysprosium atom is in a mono-capped trigonal geometry. Moreover, the bond lengths of the dysprosium and the oxygen atoms from the coordinated water molecules are 2.396 Å (Dy-O7) and 2.439 Å (Dy-O8), respectively, which are comparable to that for the protonated phenalenyl in [Dy(PLN)_2_Cl(HPLN)(EtOH)] **1** (Dy-O5) and in [Dy(PLN)_3_(HPLN)] **2a** (Dy1-O7) and that for the coordinated EtOH molecule in [Dy(PLN)_2_Cl(HPLN)(EtOH)] **1** (Dy-O7) and in [Dy(PLN)_3_(EtOH)] **2b** (Dy2-O15). Lastly, due to the presence of three water molecules, several inter- (O9H9A-O2 3.02 Å , O9H9A-O5 3.06 Å and O9H9B-O9 2.99 Å) and intra-molecular (O7H7A-O1 2.89 Å, O7H7B-O1 2.79 Å, O7H7B-O6 2.73 Å, O8H8A-O3 2.72 Å and O8H8B-O2 2.98 Å) hydrogen bonds occur in the crystal lattice (Figure S4, Supporting Information File 1).

### MALDI–TOF studies

The MALDI–TOF spectra (positive mode) of complexes **1**–**4** and the sublimed product **4’** are illustrated in Figure S5 (Supporting Information File 1). In all the spectra, the highest intensity peak corresponds to the [Dy(PLN)_2_]^+^ fragment (*m*/z = 554), in which the cation is formed by the elimination of one anionic ligand. This is an indication that all the complexes at least have two phenalenyls around the dysprosium atom. The trace peak observed at *m*/z = 572 is relative to the fragment [Dy(PLN)_2_(H_2_O)]^+^. The absorption of water molecules during the preparation of the samples is usual. However, in the spectra of the sublimed complex, which was prepared under anhydrous conditions, the relative intensity of the fragment [Dy(PLN)_2_(H_2_O)]^+^, both before and after the sublimation process, is considerably lowered. Other three small fragments in the spectra are observed for a neutral Dy(PLN)_3_ core plus a metal cation of Li^+^ or Na^+^ ([Dy(PLN)_3_Li]^+^, *m*/z = 756; [Dy(PLN)_3_Na]^+^, *m*/z = 772) and for a neutral Dy(PLN)_3_ core plus a protonated phenalenyl [H_2_PLN]^+^ cation [Dy(PLN)_3_(H_2_PLN)]^+^ (*m*/z = 945). The fact that these fragments are present in the spectra of complex **4** and its sublimed species **4’** suggests that the sublimable dysprosium complex contains three deprotonated phenalenyl ligands as structurally proposed in Scheme 2.

### NMR experiments

The paramagnetic ^1^H NMR spectra of complexes **1**–**4** obtained in deuterated DMSO are shown in Figure S6 (Supporting Information File 1). The set of resonances between 16 and 6 ppm in the low-ppm range of the spectrum of **1** clearly originates from the free non-deprotonated ligand. This is confirmed by comparison with the ^1^H NMR spectrum obtained for the pure ligand, which lacks two observations: (i) The peaks are not shifted to high ppm and broadened (the H–H coupling is still visible) by the paramagnetic dysprosium, and (ii) the peak at about 16 ppm is characteristic of the extremely de-shielded proton, which is involved in a strong intramolecular hydrogen bond with the α,β-conjugated carbonyl group of the phenalenyl rings. A set of peaks between 28 and 38 ppm shown in all the three spectra can be assigned to protons of the deprotonated phenalenyls, which are common to all the dysprosium complexes. However, two peaks between 40 and 50 ppm are observed only in the spectra of complexes **1** and **2**, but not for **4**, which is synthesized in the absence of EtOH. Thus these peaks are attributed to EtOH coordinated to the dysprosium in complexes **1** and **2**. In addition, the resonances between 18 and 24 ppm, which are noticeable in the spectra of complex **2** and of the sublimed species, could be assigned to the protonated ligand. Probably, they are not detected for complex **1** due to the low intensity of the spectrum, or due to a partial decomposition of the complex in DMSO. At last, the spectrum of **4** is characterized by a set of resonances between 6 and 10 ppm. These resonances cannot be assigned to unreacted free ligands, because the peaks are broadened and the characteristic peak at about 16 ppm is missing. However, they are in region of aromatic proton signals and are not shifted at high chemical shift from the paramagnetic Dy(III) ion.

### UV–vis experiments

**Figure 4:**
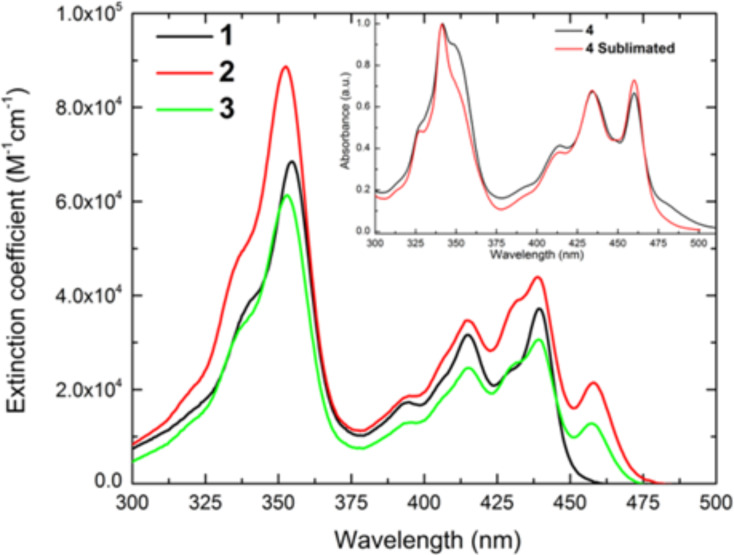
Absorption spectra of the three dysprosium complexes in diluted (2 × 10^−6^ M) DMSO solutions of **1–4** at room temperature.

To further characterize the phenalenyl-based SMMs we have measured at room temperature the absorption spectra of the diluted DMSO solutions of complexes **1**–**4** and of the sublimed product **4’**. For comparison, UV–vis spectra were recorded for the free HPLN ligand in parallel. The spectra of the three dysprosium compounds present a similar pattern, as illustrated in Figure 4. Two main absorption bands, which derive from the characteristic α,β-conjugated carbonyl group of the ligand [37], are visible: one between 375 and 475 nm and another one between 300 and 375 nm. Both absorptions take place in the ligands. The former is related to n→π* transitions, while the latter is associated to π→π* transitions. Interestingly, there is no evidence of bands arising from charge or energy transfers between ligands and metal. Due to the limited contribution of the metal, the absorption peaks, which are listed in Table 1, display minimal shifts in comparison to the free ligand. In contrast, the extinction coefficients of the dysprosium complexes are about fourfold compared to those of the free ligand. This is expected since the complexes are formed by two, three or four ligands. In addition to that, the complexes **2** and **3** are characterized by an additional peak at 457–458 nm. The origin of this peak can be ascribed to the common feature in the structures of these two complexes: Complexes **2** and **3** are both formed by a Dy(PLN)_3_ core but there is a Dy(PLN)_2_Cl core in complex **1**.

**Table 1:** List of the absorption peaks with their extinction coefficients.

compound	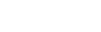	ε_λ_ (M^−1^·cm^−1^)	compound	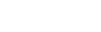	ε_λ_ (M^−1^·cm^−1^)

**9-HPLN** [38–39]	440.0 414.5 394.5 355.0	9700 8300 4600 17600	**1**	439.5 415.0 394.5 354.5	37200 31600 17294 68500
**2**	458.0 439.0 414.5 395.0 352.5	21500 43900 34700 18700 88700	**3**	457.5 439.0 415.5 395.5 353.0	12800 30600 24600 13000 61300

The correspondent maximum of 349 nm of **1**–**3** is found at 341 nm in complex **4** both before and after the sublimation. Nevertheless, the spectrum of **4**, before the sublimation, has a shoulder at 349 nm, which is less pronounced after the sublimation process. Considering that the bands between 300 and 375 nm are associated to π→π* transitions centered on the phenalenyl ligands, the shift of the bands can result from slight differences in the structures of the complexes. As a consequence of the few milligrams of material obtained by the sublimation process, we were unable to compare the extinction coefficients of the complexes. As observed previously in the absorption spectra, the ligand dominates the photophysical properties of the dysprosium complexes. As a result, the emission spectra of the three complexes are almost identical (Figure S7, Supporting Information File 1). Moreover, no sensitization of dysprosium is observed. A complete photophysical analysis is necessary to investigate the effect of the structure on the photoluminescence of those complexes.

### Magnetic studies

Both static (dc) and dynamic (ac) magnetic properties have been investigated of the complexes **1**–**3**, but not for **4**. The single crystal structure of complex **4** is not available up to now and the sublimed product **4’** has been obtained only in a small quantity. Therefore, the magnetic studies on this compound were not feasible yet.

#### Static magnetic properties

The temperature dependence of the dc magnetic susceptibility has been measured in an applied magnetic field of 1000 Oe in the temperature range between 1.8 and 300 K for all three complexes. At 300 K, the product χ*T* of **1**–**3** is 14.11, 13.56 and 14.19 cm^3^·K·mol^−1^, respectively, which are all in good agreement with the expected value of 14.17 cm^3^·K·mol^−1^ for one Dy(III) metal ion (*S* = 5/2, *L* = 5, ^6^H_15/2_, *g* = 4/3) [40]. Decreasing the temperature, the product χ*T* continuously falls to 8.78, 10.90 and 8.00 cm^3^·K·mol^−1^ at 1.8 K for **1**–**3**, respectively (see Figures in Supporting Information File 1). The gradual decrease of χ*T* vs *T* is indicative of the type of paramagnetic behavior resulting from the thermal depopulation of the Stark sublevels of the ^6^H_15/2_ ground state or of low-lying excited states of the Dy(III) ion while decreasing the temperature [41–47]. The field dependence of the magnetization at low temperatures has been measured at 2, 4 and 5 K. At 2 K and 70 kOe the magnetization approaches about 5.17 μ_B_, 5.22 μ_B_ and 6.35 μ_B_ for **1**–**3**, respectively. However, due to the incomplete saturation of the magnetization, a residual slope is observed at high fields indicating the presence of magnetic anisotropy in the material [48–49]. Moreover, no hysteresis effect is observed in all three cases under these conditions.

#### Dynamic magnetic properties

As a consequence of the presence of magnetic anisotropy, the slow relaxation of magnetization has been probed by measuring ac susceptibilities as a function of the temperature at different frequencies as well as a function of frequency at different temperatures. The plots are illustrated in Supporting Information File 1 and the results are summarized in Table 2.

**Table 2:** Magnetization dynamics of compounds **1**–**3**.

compound	χ″ in zero dc field	pre-exponential factor 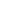 (s), energy gap Δ (K)	width of distribution α

**1**	approx. 13 K at 1500 Hz	3.3 × 10^−6^, 43.8 (0 Oe) 2.9 × 10^−6^, 49.4 (200 Oe)	0.073–0.279 0.111–0.471
**2**	no signal	7.1 × 10^−6^, 14.1 (1500 Oe) 3.0 × 10^−5^, 7.6 (3000 Oe)	0.179–0.476 0.266–0.368 (3.0–5.5 K)
**3**	no maxima	2.3 × 10^−4^ (0 Oe) 2.0 × 10^−6^, 36.5 (500 Oe)	0.103–0.179 0.117–0.468

**Figure 5:**
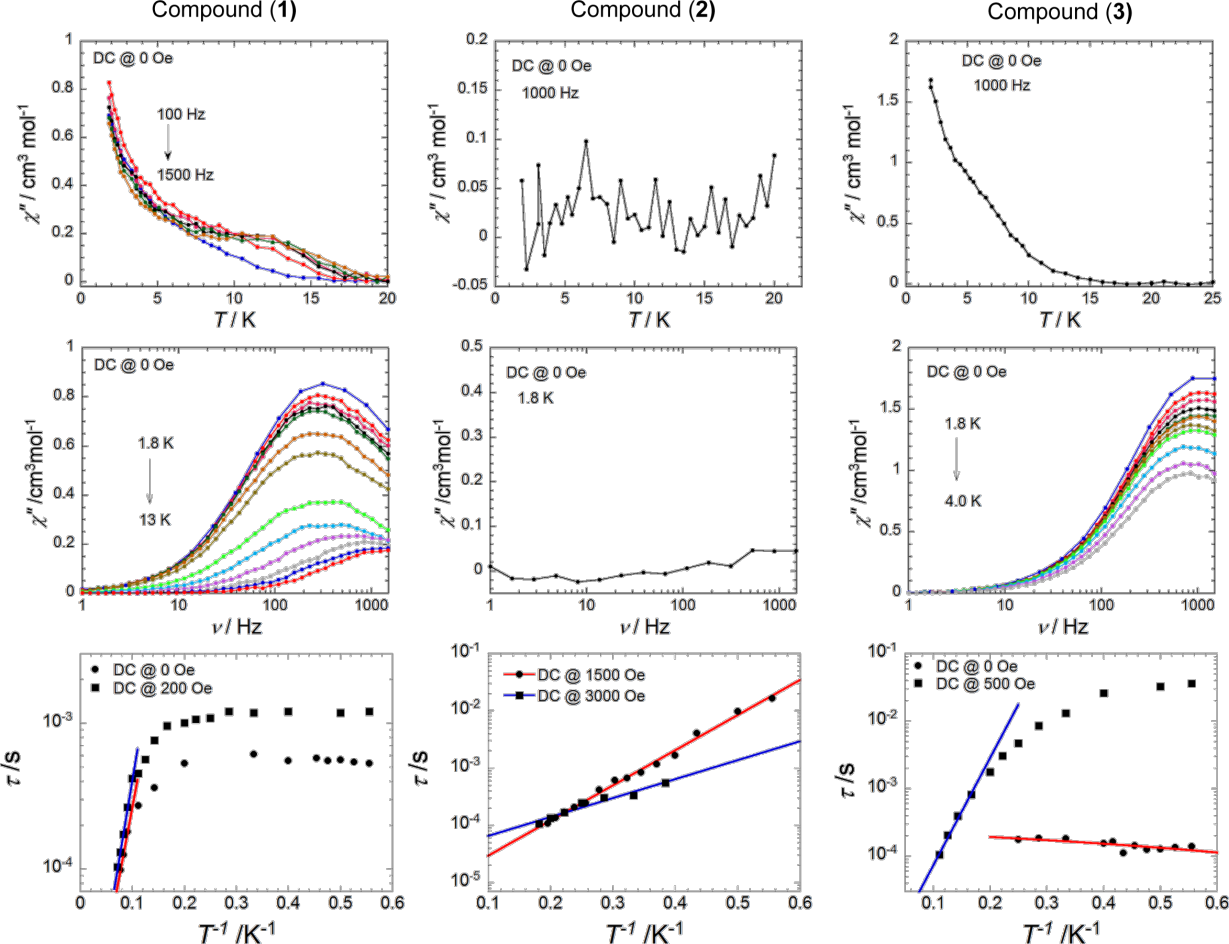
(Top) Temperature dependence of out-of-phase component of ac magnetic susceptibility under zero dc field. (Middle) Frequency dependence of out-of-phase component of ac magnetic susceptibility under zero dc field. (Bottom) Arrhenius semi-log plots of the relaxation time, τ vs 1/*T* from ac susceptibility measurements under a zero dc field and applied dc field. The solid lines represent a linear fit in the thermally activated range of temperature. The parameters are discussed in the text. Sample codes are indicated in the insets of the figures.

The ac susceptibilities of compound **1** under zero dc field show that a frequency-dependent in-phase and out-of-phase signal is detected below 20 K suggesting a slow relaxation of the magnetization. At a frequency of 1500 Hz, the out-of-phase component first reaches a maximum at 13 K and then steadily increases rather than declining to zero while decreasing the temperature. This indicates a transition from a thermally activated to a temperature-independent regime in the relaxation process [36,50–51]. Shape and frequency dependence of the out-of-phase component in ac susceptibilities suggests that **1** might be a SMM. The relaxation time plotted in Figure 5 was extracted with an Arrhenius law by fitting the frequency sweeping data between 11 and 13 K. In doing so, we estimated the characteristic energy gap Δ for the thermally activated relaxation process to be 43.8 K, and the respective pre-exponential factor τ_0_ to have a value of 3.3 × 10^−6^ s. Additionally, a saturation of about 5 × 10^−4^ s, relative to the quantum-tunneling process, is obtained below 5 K.

In relaxation processes of SMMs that are to a certain extent subjected to quantum effects, the application of a small dc field can remove the state degeneracy, and accordingly also the probability of quantum tunneling. Aiming to explore the relaxation process and to evaluate the quantum tunneling effect, the frequency dependence of the ac susceptibility was estimated at 1.8 K under a small external dc field. The characteristic frequency for compound **1** is 315 Hz at 1.8 K under zero field, whereas it decreases to 180 Hz under a dc field of 200 Oe. Thus, similar to what has been observed for some SMMs earlier, a small external dc field indeed slows down the relaxation due to the suppressed quantum tunneling of the magnetization (QTM) [52].

Lastly, ac susceptibilities as a function of the temperature have been measured under a dc field of 200 Oe to estimate the effective relaxation time (Figure 5). The data were fitted with an Arrhenius law function in the temperature range between 10 and 14 K. The characteristic SMM energy gap Δ is 49.4 K and the pre-exponential factor is τ_0_ is 2.9 × 10^−6^ s. Additionally, a quantum relaxation time of 1 × 10^−3^ s is observed below 7 K. Compared to the data calculated in zero-field, the energy gap Δ and its corresponding pre-exponential factor τ_0_ are fairly similar, suggesting that the quantum tunneling effect in **1** is not pronounced. However, the quantum relaxation time at low temperatures under 200 Oe is twice as high as that obtained under zero dc field.

For compound **2**, at a zero dc field, no out-of-phase component of ac susceptibility was detected at 1000 Hz suggesting the possible presence of an energy barrier to the relaxation of the magnetization, but it is short-cut by a fast tunneling relaxation process at zero dc field (Figure 5). In such a case, ac susceptibility measurements in the presence of a weak dc field could slow down the tunneling process which enables one to further investigate the dynamic magnetic properties of **2**. Indeed compound **2** shows a field-induced slow relaxation of the magnetization. The intensity of the out-of-phase component of the ac susceptibility is dramatically increased when a dc field is applied confirming the cooperation between a slow magnetic relaxation and a rapid quantum tunneling. The relaxation process immediately slows down to 40Hz when the applied field reaches 500 Oe, in contrast, it becomes faster at ca. 10 Hz up to 1500 Oe. This behavior points to the fact that this compound, concerning its relaxation dynamics, is characterized by a very fast tunneling process at zero dc field.

When the applied dc field is increased from 1500 Oe upwards, the relaxation process oscillates to a higher frequency and subsequently slows down again at 3000 Oe. This observation implies that there is more than one relaxation process in the system. That is not surprising if we correlate this behavior with the X-ray crystal structure of this compound. As there are two isolated Dy(III) centers present in this compound, the presence of multiple relaxation processes is likely to be correlated to the different individual ion anisotropies around the two Dy(III) centers. However, it is not possible to distinguish them based on the present data.

Then, the frequency sweeping ac susceptibility measurements are performed under a dc field of 1500 and 3000 Oe, respectively. Under a dc field of 1500 Oe, one set of peaks is observed in the out-of-phase component of the ac susceptibility. Conversely, under a dc field of 3000 Oe, two sets of peaks are clearly visible in the plot of the frequency dependence of the out-of-phase component of the ac susceptibility, indicating the presence of more than one relaxation pathway. Moreover, as a stronger dc field is applied, the smaller peak at lower frequencies increases at the expense of the larger peak beyond the window of the measurements. The energy barrier Δ and the pre-exponential factors τ_0_ of the relaxation pathways are calculated by plotting the relaxation time τ vs 1*/T* (Figure 5). At 1500 Oe, the relaxation time deduced from the data between 1.8 and 5.1 K approximately follows an activated behavior with an energy gap Δ of 14.1 K and a pre-exponential factor τ_0_ of 7.1 × 10^−6^ s. At 3000 Oe, the relaxation time Δ of the relaxation pathway located at higher frequencies in the temperature range between 2.6 and 5.5 K is 7.6 K and its pre-exponential factor τ_0_ is 3.0 × 10^−5^ s. The characteristic parameters obtained under a dc field of 1500 Oe and 3000 Oe are roughly in the same order of magnitude, indicating that the mechanism of the two relaxation processes, corresponding to the two isolated Dy(III) ions in **2**, is most probably the same.

The dynamics of the magnetization of compound **3** was studied by the same methods applied for compounds **1** and **2** described as above. The ac out-of-phase component is clearly observed up to 20 K under zero dc field, but no maximum could be observed in the χ″ component indicating that the blocking temperature is below 1.8 K. As demonstrated in Supporting Information File 1, the relaxation time retains its value of ca. 2.3 × 10^−4^ s, being nearly temperature-independent between 1.8 K and 4.0 K. Above 1.8 K, the constraint set by the low-temperature limit of our magnetometer, the peaks of χ″ signals could only be detected in the frequency range above 1000 Hz. This phenomenon is attributable to temperature-independent zero-field fast quantum tunneling of the magnetization, whereby the degeneracy of the *m*_S_ states can be removed and the probability of the zero-field QTM between the ±*m*_S_ states lowered by the effect of a weak external field [52]. Then the frequency dependence of the ac susceptibility data at 1.8 K has been studied by applying a small dc field up to 3 kOe. As expected, the zero-field QTM is partially suppressed. A field of 500 Oe is applied to investigate the frequency and temperature dependence of the ac susceptibility leading to a relaxation time Δ of the relaxation pathway in the temperature range between 7 and 10 K of 36.5 K and its pre-exponential factor τ_0_ of 2.0 × 10^−6^ s. Below 7 K, the relaxation time increases non-exponentially and is substantially curved. This curvature indicates that the moment of **3** has access to multiple pathways for spin reversal, which means that the Orbach thermally activated relaxation process and quantum-tunneling process (Raman or direct processes) coexist in this temperature regime.

## Discussion

**Figure 6:**
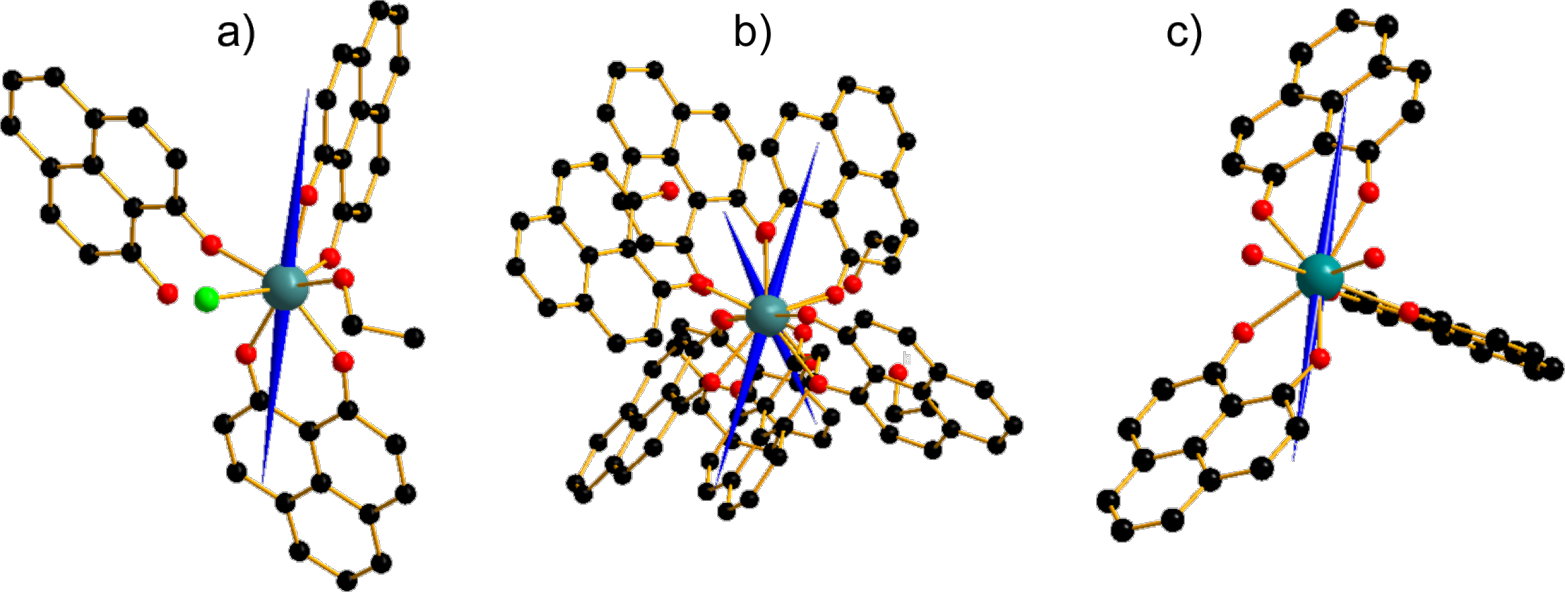
Orientation of the main anisotropy axis in complexes **1**–**3** indicated as blue arrows (a, b and c) calculated using the Magellan Software. Coordinates are taken from the crystal structures depicted in Figures 1–3.

The program Magellan [53] was used to extract information about the magnetic easy axis in complexes **1**–**3** (Figure 6). On the basis of the Magellan output, we found that in all three cases the axis of preferred alignment extends along with the planes of deprotonated phenalenyls defined by the aromatic rings. The co-ligands such as the Cl^−^ anion, H_2_O and EtOH molecules act as weak ligands that interact with the dysprosium ion in the hard plane where the biggest contribution to tunneling would be expected. The weak ligand fields imposed on the dysprosium ion by these co-ligands lead to non-negligible transverse components that induce the quantum tunneling effect so as to be suppressed with the application of external dc field. On the other hand, the presence of these co-ligands in these systems reduces the three-fold symmetry of the whole molecule. Indeed, low-symmetry elements present in such a ligand field play an important role in facilitating tunneling or other magnetic relaxation processes [54]. This effect is consistent with the observation of true thermally activated relaxation resulting in a highly curved relaxation time down to the low temperature regime (Figure 5). The foregoing results obtained from this calculation well explain the tunneling dynamics of magnetization discussed in the earlier section. Finally, it is worthwhile to point out that the anisotropy axes of two dysprosium atoms in compound **2** are symmetrically twisted in a torsion angle of 40.7° (Figure 6b and Supporting Information File 1). As mentioned in the structural description, this molecule is rich of hydrogen bonding and the π–π stacking. With such a narrow torsion angle calculated from Magellan, one can think that the two magnetic centers could be strongly interacting through the π orbitals of the condensed rings so that the single-ion anisotropy between the two molecules is probably canceled out leading to a zero overall anisotropy so that no out-of-phase signal could be observed under zero dc field. In principle the effect of the intermolecular interaction could be evaluated by preparing a diamagnetically doped system, but it is too much work compared to the importance of the information that can be extracted.

**Figure 7:**
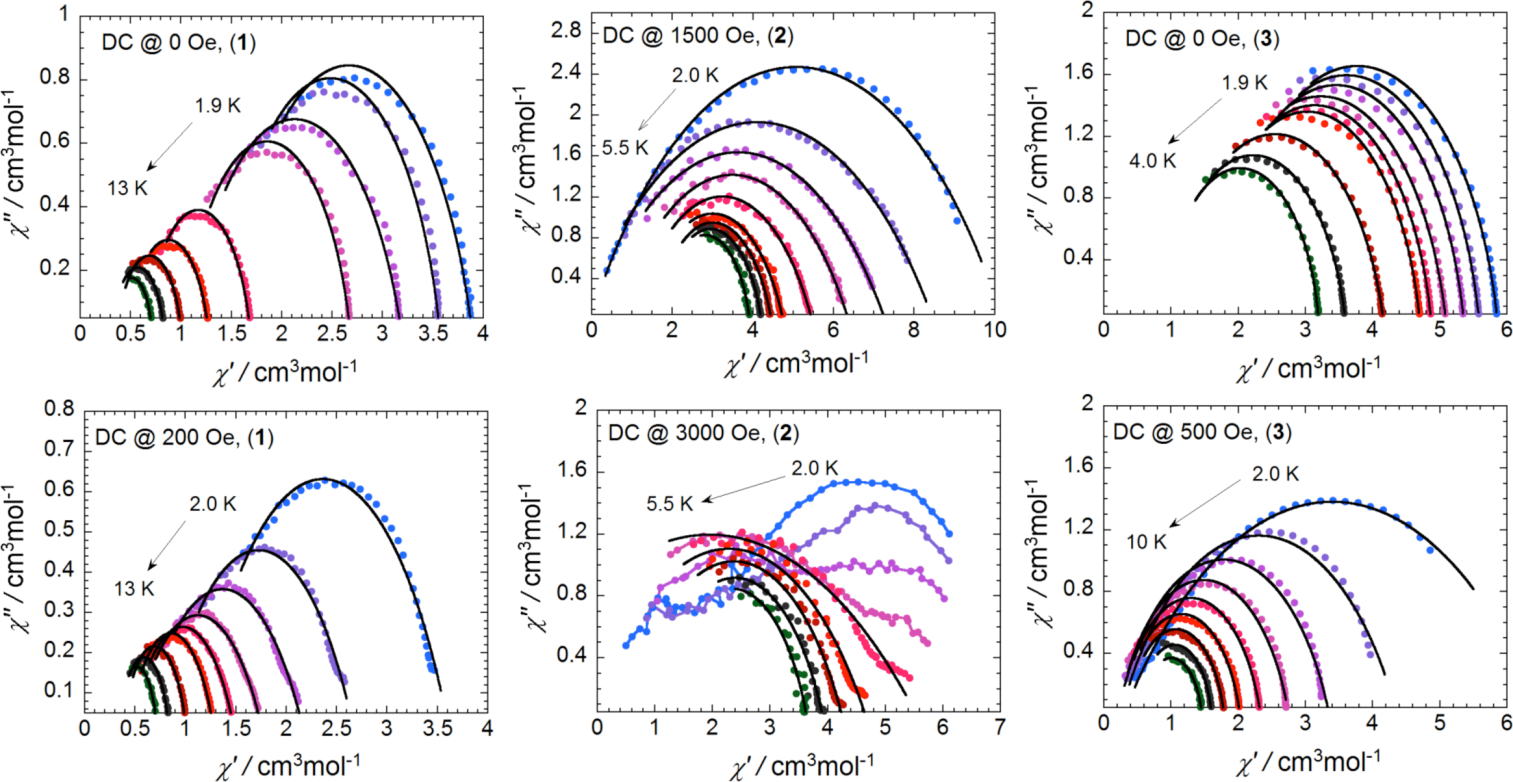
Cole–Cole plots under zero or different dc fields in the given temperature ranges. Sample codes are indicated in the graphs. The black solid lines represent the least-squares fit obtained with a generalized Debye model. The parameters are discussed in the text. In the figure for **2** under a dc field of 3000 Oe, the colored solid lines are guided to eyes.

To see if it is possible to further study the relaxation process and to better characterize the time distribution for relaxation, a Cole–Cole plot of the out-of-phase vs in-phase susceptibilities at low temperatures was constructed. The width of distribution quantified by a parameter α indicates how significantly the relaxation process is distributed, i.e., when a single relaxation process is active, a semicircular shape would be anticipated in the Cole–Cole plot with the α being close to zero. The χ″ vs χ′ data (Figure 7) under zero or dc different fields were fitted by the extended Debye Model [55]. All derived parameters are summarized in Supporting Information File 1, Tables S2–S7. For compound **1**, under zero dc field, the width of distribution α varies from 0.073 to 0.279 in the entire temperature range of 1.8–13 K. From 7.0 to 13 K, the small α values below 0.16 are compatible with the SMM behavior. The process within this observed temperature range is thermally activated and leads to the exponential temperature dependence of the relaxation time of 3.3 × 10^−6^ s, which is a characteristic value observed for typical SMMs [56–57]. Under a dc field of 200 Oe, α varies from 0.111 to 0.471 between 1.8 and 13 K. Below 6 K, α is down to the range of 0.400–0.471 suggesting that there is likely to be more than one relaxation process operating at these temperatures. Indeed, as seen from Figure 5, a transition from a thermally activated to a temperature-independent regime is detected in the relaxation rates. For compound **2**, under a dc field of 1500 Oe, the width of distribution α varies from 0.179 to 0.476 up to 5.5 K but is not greater than 0.366 above 3.9 K. However, under a dc field of 3000 Oe, two obvious sets of Argand plots corresponding to two relaxation processes are observed below 3.5 K, which is consistent with the experimental results discussed in the dynamic properties of **2**. With the increase of temperature up to 5.5 K, the width of distribution α is determined to be 0.266–0.368. Within this temperature range the relaxation time obeys the Arrhenius law. For compound **3**, the relaxation rate under zero field is almost independent from the temperature indicating that the relaxation process remains in the quantum tunneling regime with a quantum time of 2.3 × 10^−4^ s. The width of distribution is very narrow with α of 0.103–0.179. Under a dc field of 500 Oe, the relaxation slows down so that it could be detected up to a high temperature of 9 K. The width of distribution varies from 0.117 to 0.468, which is very similar to the values obtained for compound **1** under a dc field of 200 Oe. Again the relaxation process encounters a crossover from a thermally activated (4.0–10 K) to a temperature-independent regime (1.8–4.0 K).

## Conclusion

Three phenalenyl-based mononuclear dysprosium complexes have been synthesized from the reaction of 9-hydroxy-1*H*-phenalen-1-one with hexahydrous DyCl_3_ in a 3:1 molar ratio in the presence of NaH or diisopropylamine. Single crystals in reasonable yields were obtained by slow evaporation of the solvents. All compounds were characterized by standard spectroscopic and analytical techniques and their solid-state structures were established by single crystal X-ray crystallography.

The magnetic properties of complexes **1**–**3** were studied in detail suggesting that these complexes are SMMs with fast zero-field quantum tunneling of the magnetization. The presence of co-ligands reduces the three-fold symmetry of the molecules so that non-negligible transverse anisotropy is imposed on the single dysprosium ion. The relaxation processes present in these complexes were discussed elaborately with the analysis of the width of distribution in the Cole–Cole plots. The energy barriers and its corresponding pre-exponential factors associated with the relaxation processes are determined under both zero and external dc fields. In particular, the relaxation process of **1** under zero dc field is a characteristic of an energy gap Δ of 43.8 K and a pre-exponential factor τ_0_ of 3.3 × 10^−6^ s.

Furthermore, a sublimable phenalenyl-based dysprosium complex **4** has been made by implementing a synthetic strategy under anhydrous conditions. The sublimed species **4’** was preliminary characterized, confirming its thermal stability. However, growing single crystals of these complexes is fundamental to elucidate their exact structures so as to study their magnetic properties. There are two concerns regarding the exploration of this system. First of all, these molecules can be grafted by sublimation onto surfaces for a variety of studies, which further offers the potential to be used for device. Another important point is that a higher blocking temperature could be expected in compound **4** than those observed in compounds **1**–**3** as a consequence of the removal of coordinated solvents. In the cases of **1**–**3**, the Magellan output reveals that the presence of coordinated solvents or anions invokes a transverse anisotropy facilitating tunneling pathways. Based on the design criteria proposed by Chilton et al. [58–59] for the two-coordinate complexes of Dy(III), large values of the effective energy barrier, *U*_eff_, are immediately diminished if axial ligation is disrupted by solvent coordination. Considering this assumption, an increase of the magnetic relaxation barrier so as to blocking temperatures could be expected if such complexes could be obtained in the absence of a coordinating solvent. Therefore, our further efforts will be to isolate and structurally characterize the complexes **4** and **4’** and their respective magnetic properties, in particular after being sublimed onto surfaces. This work is currently in progress. During the submission of this work, a few interesting Dy(III)-SIMs with *D*_5_*_h_* symmetry have been reported [60–62]. The three examples all show a fascinating anisotropic barrier over 500 K as a result of the higher order symmetry around Dy(III) ion. This is related to the concept that we discussed in our introduction.

## Experimental

### Instrumentation

Elemental analysis of carbon and hydrogen, were carried out in a Vario Micro Cube. Infrared spectra were recorded using KBr pressed pellets with a Perkin-Elmer Spectrum GX FTIR spectrometer (MAGNA FTIR 750, Nicolet) in the region of 4000–400 cm^−1^ region. Paramagnetic ^1^H NMR spectra were recorded in a Bruker FT-NMR Avance III 500 MHz with deuterated solvents as internal standards. Matrix-assisted laser desorption/ionization-time of flight (MALDI–TOF) mass spectrometric data were acquired on a MALDI–TOF Synapt G2-S HDMS without additional matrix compound other than the sample itself. The absorption spectra were acquired at room temperature for diluted (2.0 × 10^−6^ M) DMSO (spectrophotometric grade) solutions on a Cary 500 Scan UV–vis–NIR spectrophotometer using a 1 cm quartz cell. Emission spectra were recorded at room temperature for diluted (2.0 × 10^−6^ M) DMSO (spectrophotometric grade) solutions on a Cary Eclipse Fluorescence spectrophotometer using a 1 cm quartz cell.

### X-ray crystallography

Single crystal X-ray diffraction data were collected on a STOE IPDS II diffractometer with graphite monochromated Mo Kα radiation (λ = 0.71073 Å). Structure solution and refinement against *F*^2^ were carried out using shelxs and shelxl software. Refinement was performed with anisotropic temperature factors for all non-hydrogen atoms (disordered atoms were refined isotropically); hydrogen atoms were calculated on idealized positions. Crystallographic data are summarized in Table S1 (Supporting Information File 1).

### Magnetic studies

Magnetic measurements were obtained with a Quantum Design SQUID magnetometer MPMS-XL. Direct current (dc) susceptibility measurements were carried out over the temperature range of 1.8–300 K under an applied dc field of 1000 Oe. Magnetization measurements were made at low temperatures with applied dc fields from 0 to 70 kOe. Alternating current (ac) susceptibility measurements were measured under zero dc field with an oscillating ac field of 3 Oe and ac frequencies ranging from 1 to 1500 Hz. Measurements were performed on polycrystalline samples. The ground powder was restrained in Apiezon grease. The magnetic data were corrected for the sample holder.

### Synthesis

All the purchased chemicals and solvents were used as received without any further purification unless otherwise stated. The reactions involving water-sensitive materials were carried out with distilled THF (over Na) and implementing standard Schlenck techniques; the glassware was kept in an oven at 80 °C, evacuated and flushed with argon prior to use.

#### Synthesis of [Dy(PLN)_2_(HPLN)Cl(EtOH)] (**1**)

To a yellow solution of 9-hydroxy-1*H*-phenalen-1-one (117.7 mg, 0.6 mmol, 3 equiv) of CHCl_3_ (5 mL) was added dropwise a colorless solution of NaH (14.4 mg, 0.6 mmol, 3 equiv) in ethanol, leading to a yellow suspension. After stirring for 5 min, a solution of DyCl_3_·6H_2_O (75.4 mg, 0.2 mmol, 1 equiv) in ethanol (15 mL in total) was added dropwise to afford a homogeneous solution, which was left to stir at room temperature for 2 h and then brought to reflux for 3 h. The reaction solution was then left to stir overnight at room temperature. After filtration, the filtrate was allowed to evaporate slowly. Red blocks were formed over two weeks. Yield (single crystals) based on Dy: 20.8 mg, 13%. Elemental analysis (%) calculated (C_41_H_28_ClDyO_7_, 830.58 g/mol): Anal. calcd for C_41_H_28_ClDyO_7_: C, 59.29; H 3.40; found: C, 59.87; H 3.67; FTIR (KBr) ν (cm^−1^): 3369, 2924, 1631, 1583, 1561, 1521, 1482, 1428, 1346, 1257, 1241, 1181, 1152, 1047, 986, 960, 851, 806, 745, 694, 644, 553, 483, 451, 432; MALDI–TOF (CH_2_Cl_2_, positive) *m*/*z* (%): 554 ([M-HL-Cl-C_2_H_5_O]^+^, 100); ^1^H NMR (500 MHz, DMSO, δ) 49.23, 40.63, 36.40, 33.58, 28.49, 16.27, 8.33, 7.77 ppm.

#### Synthesis of [Dy(PLN)_3_(HPLN)] (**2a**)·[Dy(PLN)_3_(EtOH)]·2EtOH (**2b**)

To a yellow suspension of 9-hydroxy-1*H*-phenalen-1-one (117.7 mg, 0.6 mmol, 3 equiv) of ethanol (5 mL) was added dropwise a colorless solution of diisopropylamine (60.6 mg, 0.6 mmol, 3 equiv) in ethanol, leading to no obvious color change. After stirring for 5 min, a solution of DyCl_3_·6H_2_O (75.4 mg, 0.2 mmol, 1 equiv) in ethanol (20 mL in total) was added dropwise, in which the reaction mixture remained as a yellow suspension, which was left it to stir at room temperature for 2 h and then brought it to reflux for 3 h. The reaction mixture was then left to stir overnight at room temperature. Upon filtration, the yellow filtrate was allowed to evaporate slowly. Red needles are formed over two weeks. Yield (single crystals) based on Dy: 18.3 mg, 10%. Elemental analysis (%) calculated (C_194_H_136_O_34_Dy_4_, 3661.03 g/mol): Anal. calcd for C_194_H_136_O_34_Dy_4_: C, 63.64; H 3.74; found: C, 63.51; H 3.66; FTIR (KBr) ν (cm^−1^): 3432, 3047, 2969, 1627, 1582, 1560, 1522, 1421, 1412, 1345, 1252, 1241, 1222, 1177, 1140, 1045, 985, 957, 851, 804, 745, 698, 643, 545, 482, 450, 419; MALDI–TOF (CH_2_Cl_2_, positive) *m*/*z* (%): 554 ([M − HL − L]^+^, or [M − L − C_2_H_5_O]^+^, 100).

#### Synthesis of [Dy(PLN)_3_(H_2_O)_2_]·H_2_O (**3**)

This complex is synthesized based on the same procedure as that of preparation of complex **2**. The modification is that the volume of ethanol used in this reaction is scaled up from 20 to 30 mL. The crystals of **3** as red/orange needles were grown from the slow evaporation of the yellow filtrate. Yield (single crystals) based on Dy: 12.1 mg, 8%. Elemental analysis (%) calculated (C_39_H_27_DyO_9_, 802.10 g/mol): Anal. calcd for C_39_H_27_DyO_9_: C, 58.40; H, 3.39; found: C, 58.69; H, 3.43; MALDI–TOF (CH_2_Cl_2_, positive) *m*/*z* (%): 554 ([M − L − 2H_2_O]^+^, 100).

#### Synthesis of complex **4** in anhydrous conditions [63]

A solution of hexamethyldisilazide (16.5 mL, 78.0 mmol) in 40 mL of freshly distilled THF was cooled to 0 °C. To the ice-cold solution *n*-butyllithium (1.6 M in hexane, 50.0 mL, 80.0 mmol) was slowly added and the mixture was stirred for 1 h. Then anhydrous DyCl_3_ (6.45 g, 24.0 mmol) was carefully added and the mixture was allowed to warm up to room temperature overnight under stirring. The resulting mixture was then purified by sublimation in high vacuum (2 × 10^−6^ mbar, 90 °C). Subsequently, a solution of HPLN (0.46 g, 2.4 mmol) in 20 mL in freshly distilled THF was added to the sublimed product (0.50 g, 0.8 mmol). The mixture was further refluxed at 65 °C overnight. The yellow precipitate was filtered and washed with fresh THF (0.28 g).

### X-ray crystallographic studies of **1**–**3**

Suitable crystals of compounds **1**–**3** were covered with perfluoroether oil and mounted onto a glass fiber. The crystal was transferred directly to the −93 °C N_2_ cold stream of a Stoe IPDS 2T diffractometer. Data were corrected for absorption effects using indexed faces of the crystals [64].

All structures were solved by using the program SHELXS-97 [65]. The remaining non-hydrogen atoms were located from successive difference Fourier map calculations. The refinements were carried out by using full-matrix least-squares techniques on *F*^2^, minimizing the function (*F*_o_ − *F*_c_)^2^, where the weight is defined as 4*F*_0_^2^/2(*F*_o_^2^) and *F*_o_ and *F*_c_ are the observed and calculated structure factor amplitudes using the program SHELXL-97 [63]. The hydrogen atom contributions of all compounds were calculated, but not refined. The locations of the largest peaks in the final difference Fourier map calculation as well as the magnitude of the residual electron densities in each case were of no chemical significance. The graphics of crystal structures were generated by PLATON software.

#### Crystal data of **1**

C_41_H_28_ClDyO_7_, *M* = 830.58 g/mol, triclinic, *a* = 10.4482(7) Å, *b* = 12.5823(8) Å, *c* = 13.2433(8) Å, α = 93.446(5)°, β = 112.211(5)°, γ = 95.762(5)°. *V* = 1594.55(18) Å^3^, *T* = 180.15 K, space group *P*−1, *Z* = 2, 10913 reflections measured, 5790 unique (*R*_int_ = 0.0169), which were used in all calculations. The final *R*_1_ values were 0.0172. The final *wR*(*F*^2^) was 0.0441 (all data). The goodness of fit on *F*^2^ was 1.047.

#### Crystal data of **2**

C_194_H_136_O_34_Dy_4_, *M* = 3661.03 g/mol, triclinic, *a* = 11.0591(8) Å, *b* = 17.2521(8) Å, *c* = 19.8266(10) Å, α = 92.328(4)°, β = 104.285(5)°, γ = 95.206(5)°. *V* = 3642.9(4) Å^3^, *T* = 180.15 K, space group *P−*1, *Z* = 1, 29552 reflections measured, 13592 unique (*R*_int_ = 0.0444), which were used in all calculations. The final *R*_1_ values were 0.0336. The final *wR*(*F*^2^) was 0.0639 (all data). The goodness of fit on *F*^2^ was 0.914.

#### Crystal data of **3**

C_39_H_27_O_9_Dy, *M* = 802.10 g/mol, monoclinic, *a* = 29.9539(9) Å, *b* = 10.5596(2) Å, *c* = 22.9909(6) Å, β = 120.106(2)°, *V* = 6291.0(3) Å^3^, *T* = 180.15 K, space group *C*2/*c*, *Z* = 8, 17247 reflections measured, 5879 unique (*R*_int_ = 0.0289), which were used in all calculations. The final *R*_1_ values were 0.0349. The final *wR*(*F*^2^) was 0.0813 (all data). The goodness of fit on *F*^2^ was 1.046.

#### CCDC deposit

Crystallographic data (excluding structure factors) for the structures reported in this paper have been deposited with the Cambridge Crystallographic Data Centre as a supplementary publication no. CCDC-1055828 (**1**), 1055829 (**2**) and 1055830 (**3**). Copies of the data can be obtained free of charge on application to CCDC, 12 Union Road, Cambridge CB21EZ, UK (fax: (+(44)1223-336-033; email: deposit@ccdc.cam.ac.uk).

## Supporting Information

File 1 Additional experimental data.
