# Supplementary material for: Phenalenyl-based mononuclear dysprosium complexes
Source: Beilstein J Nanotechnol. 2016 Jul 8;7:995–1009. doi: 10.3762/bjnano.7.92 (PMC4979880; doi:10.3762/bjnano.7.92)
Supplement: File 1 — Additional experimental data. [file Beilstein_J_Nanotechnol-07-995-s001.pdf]

Supporting Information

for

Phenalenyl-based mononuclear dysprosium complexes

Yanhua Lan*[1,2], Andrea Magri[1], Olaf Fuhr[1,3] and Mario Ruben*[1,4]

Address: [1]Institut für Nanotechnologie, Karlsruher Institut für Technologie (KIT), Postfach 3640, D-76344 Karlsruhe, Germany (Tel: +49 721-608-28948); [2]Institut Néel, CNRS, Nanosciences Department, BP 166, 25 rue des Martyrs, 38042 GRENOBLE Cedex 9, France; [3]Karlsruhe Nano Micro Facility (KNMF), Karlsruher Institut für Technologie (KIT), Postfach 3640, D-76344 Karlsruhe, Germany and [4]Université de Strasbourg, Institut de Physique et de Chimie des Materiaux de Strasbourg, Campus de Cronenbourg, 23 Rue du Loess, 67034 Strasbourg Cedex 2, France

Email: Yanhua Lan* - yanhua.lan@kit.edu; Mario Ruben* - mario.ruben@kit.edu

* Corresponding author

Additional experimental data

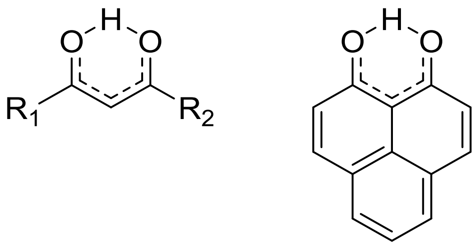

Figure S1: Chemical structure of β-diketonates and of HPLN.

X-ray crystal data

Crystal packing diagrams

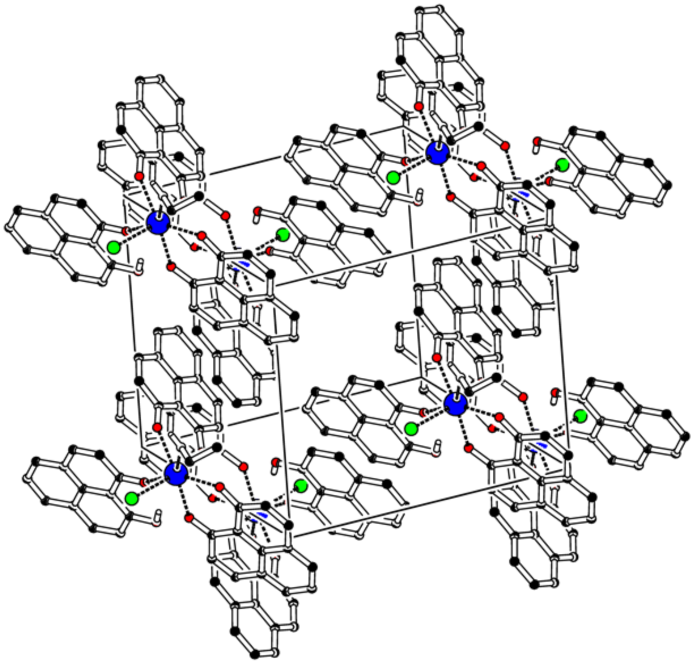

Figure S2: The molecular packing in the single crystal of **1** is illustrated in perspective view of the y–z plane.

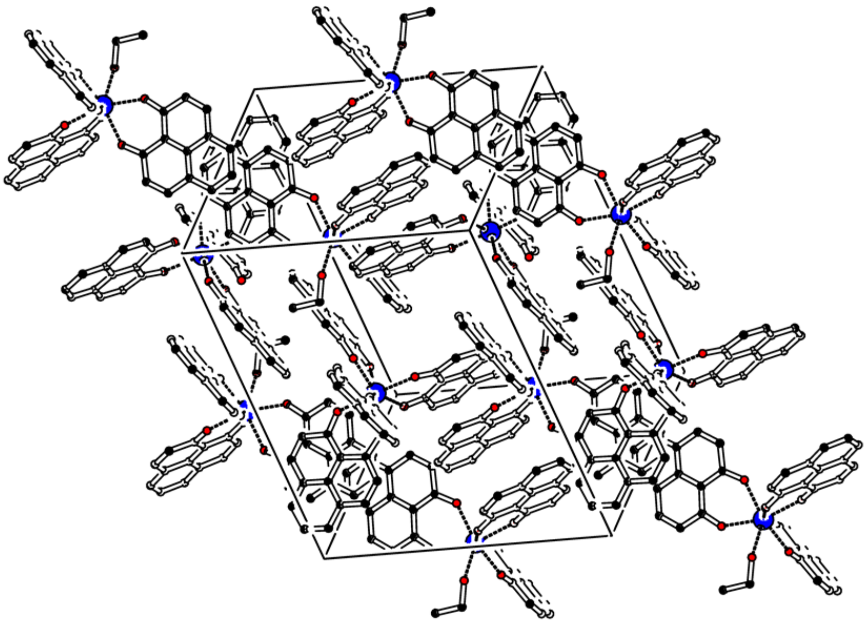

Figure S3: The molecular packing in the single crystal of **2** is illustrated in perspective view of the y–z plane.

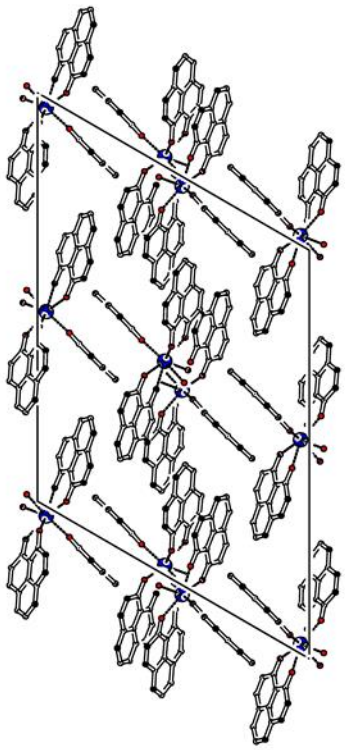

Figure S4: The molecular packing in the single crystal of [$Dy(PLN)_3(H_2O)_2$] **3** is illustrated in perspective view of the x–z plane.

Table S1: Crystal data and structure refinement for complexes **1**–**3**.

Identification code	LYH13-1 (**1**)	LYH13-2 (**2**)	LYH41-2 (**3**)
Empirical formula	$C_{41}H_{28}ClDyO_7$	$C_{194}H_{136}O_{34}Dy_4$	$C_{39}H_{27}DyO_9$
Formula weight	830.58	3661.03	802.10
Temperature/K	180.15	180.15	180.15
Crystal system	triclinic	triclinic	monoclinic
Space group	*P*−1	*P*−1	*C*2/*c*
a/Å	10.4482(7)	11.0591(8)	29.9539(9)
b/Å	12.5823(8)	17.2521(8)	10.5596(2)
c/Å	13.2433(8)	19.8266(10)	22.9909(6)
α/°	93.446(5)	92.328(4)	90
β/°	112.211(5)	104.285(5)	120.106(2)
γ/°	95.762(5)	95.206(5)	90
Volume/Å^3	1594.55(18)	3642.9(4)	6291.0(3)
Z	2	1	8
ρ_{calc}mg/mm^3	1.730	1.669	1.694
m/mm^{-1}	2.483	2.114	2.436
F(000)	826.0	1836.0	3192.0
Crystal size/mm^3	0.18 × 0.15 × 0.14	0.24 × 0.15 × 0.11	0.34 × 0.28 × 0.03
Radiation	Mo Kα (λ = 0.71073)	Mo Kα (λ = 0.71073)	Mo Kα (λ = 0.71073)
2Θ range for data collection	4.28 to 51.2°	4.28 to 51.28°	4.28 to 51.296°
Index ranges	-12 ≤ h ≤ 12, -15 ≤ k ≤ 15, -16 ≤ l ≤ 16	-13 ≤ h ≤ 13, -20 ≤ k ≤ 20, -22 ≤ l ≤ 24	-36 ≤ h ≤ 34, -11 ≤ k ≤ 12, -27 ≤ l ≤ 27
Reflections collected	10913	29552	17247
Independent reflections	5790 [R_{int} = 0.0169, R_{sigma} = 0.0170]	13592 [R_{int} = 0.0444, R_{sigma} = 0.0628]	5879 [R_{int} = 0.0289, R_{sigma} = 0.0202]
Data/restraints/parameters	5790/0/461	13592/4/1059	5879/3/453
Goodness-of-fit on F^2	1.047	0.914	1.046
Final R indexes [I>=2σ (I)]	R_1 = 0.0172, wR_2 = 0.0441	R_1 = 0.0336, wR_2 = 0.0639	R_1 = 0.0349, wR_2 = 0.0813
Final R indexes [all data]	R_1 = 0.0194, wR_2 = 0.0447	R_1 = 0.0643, wR_2 = 0.0712	R_1 = 0.0403, wR_2 = 0.0837
Largest diff. peak/hole / e Å^{-3}	0.64/-0.69	0.55/-0.85	0.97/-2.04
CCDC	1055828	1055829	1055830

Maldi-ToF

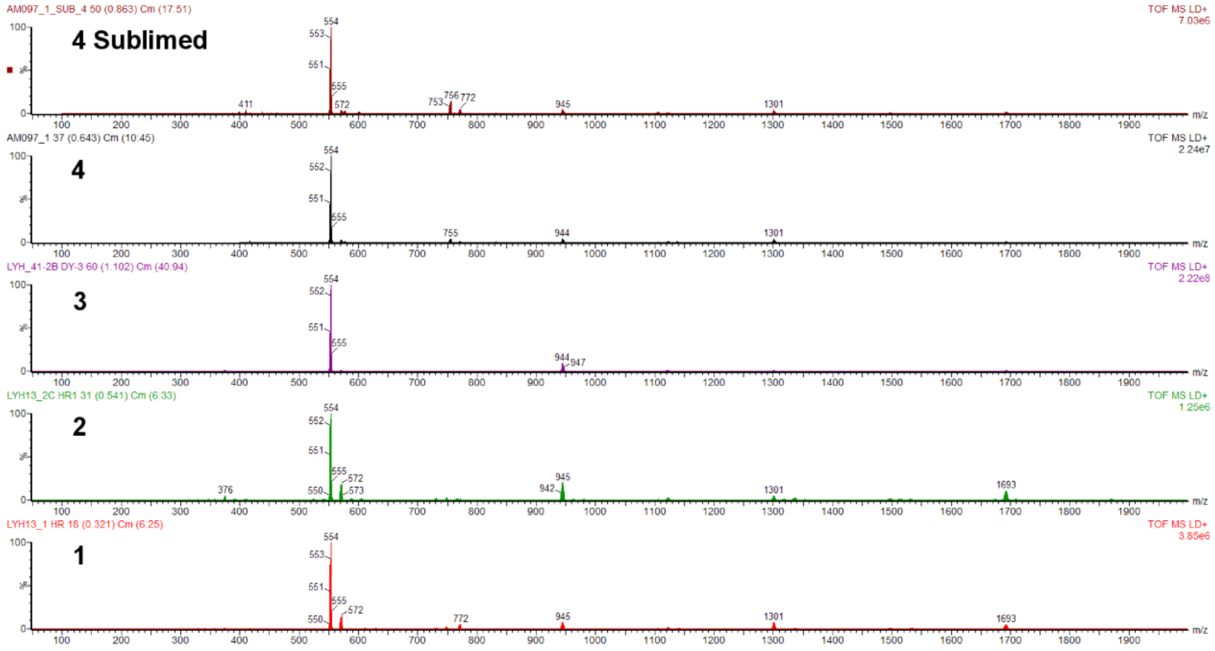

Figure S5: MALDI-TOF spectra of all complexes **1**–**3** and **4** before and after sublimation.

NMR spectra

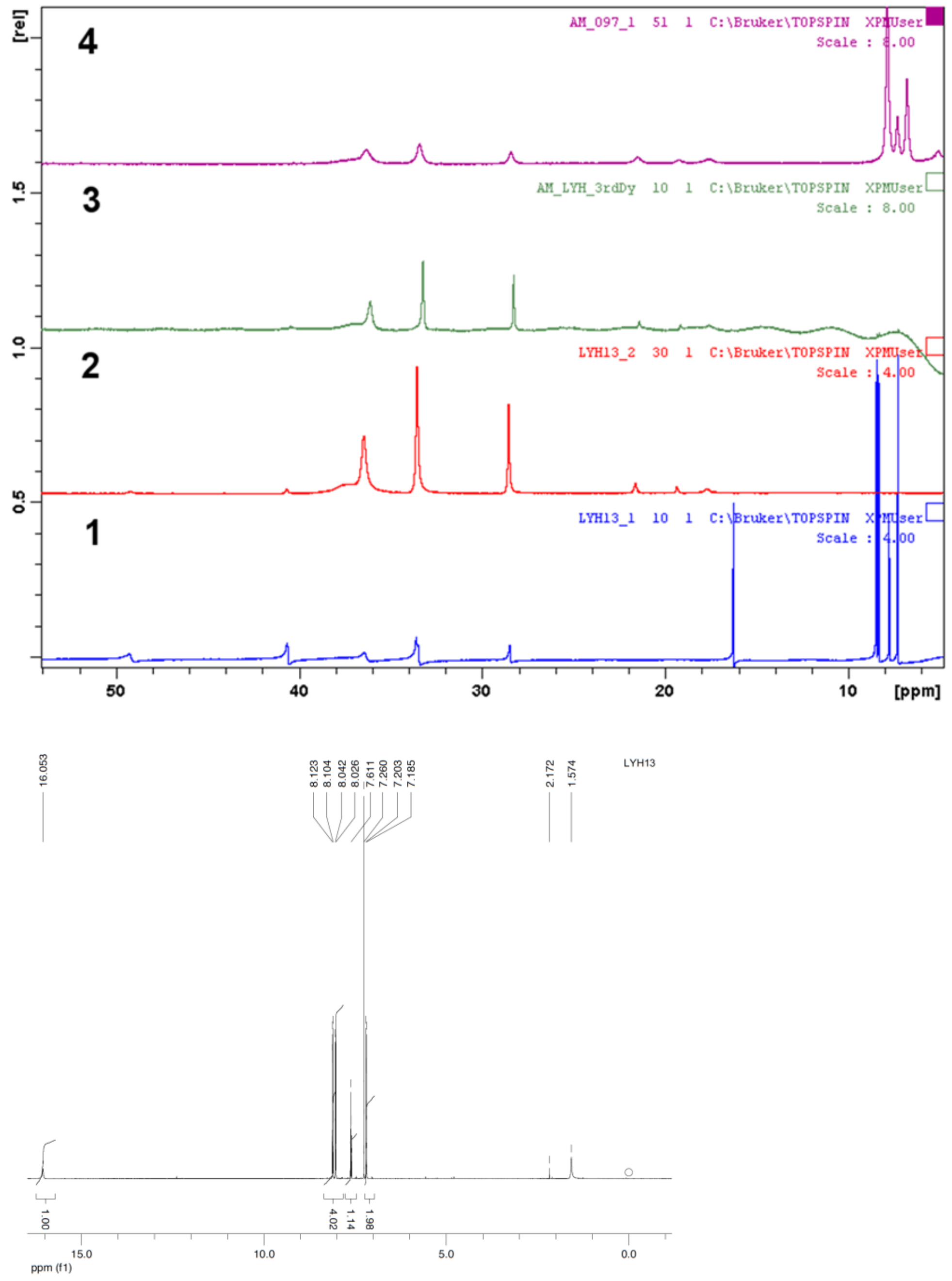

Figure S6: ^{1}H NMR spectra of compounds **1**–**4** in deuterated DMSO. The NMR spectrum of the pure ligand (bottom) is shown for reference.

UV–vis spectra

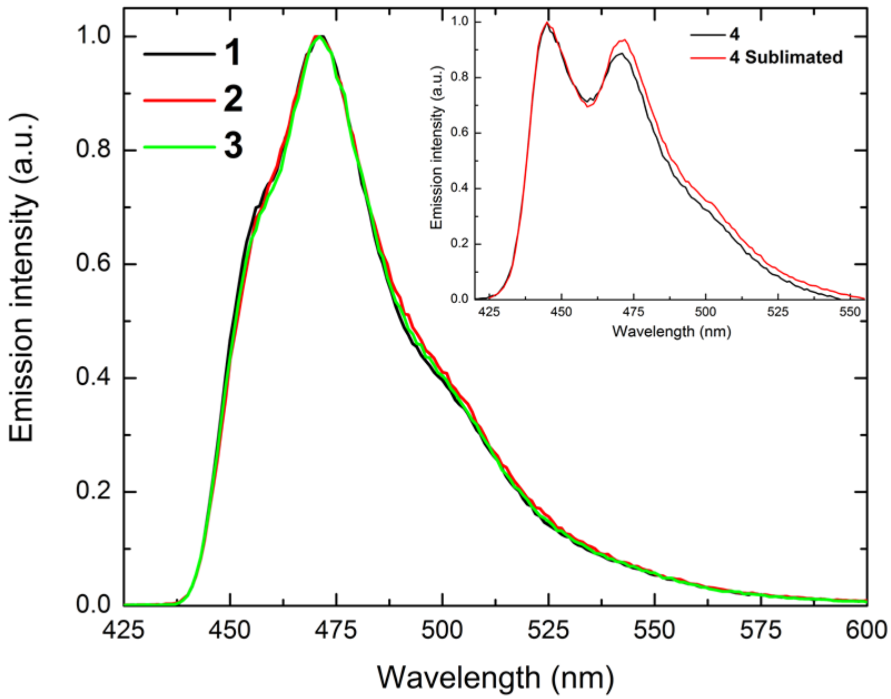

Figure S7: Emission spectra of compounds **1**–**4** in DMSO.

Magnetic data

Lyh13_1 [DyL$_2$(HL)·Cl·EtOH] – compound 1

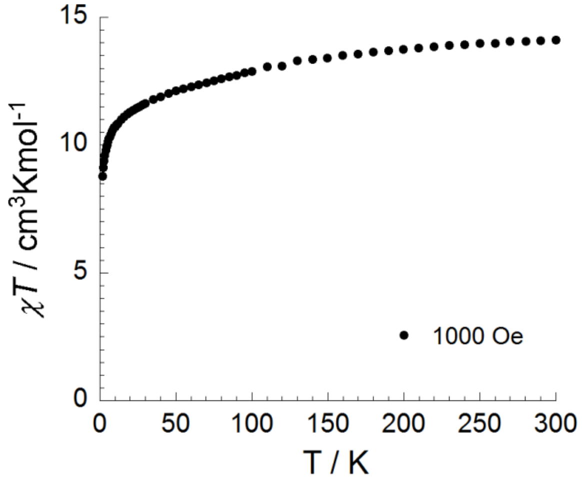

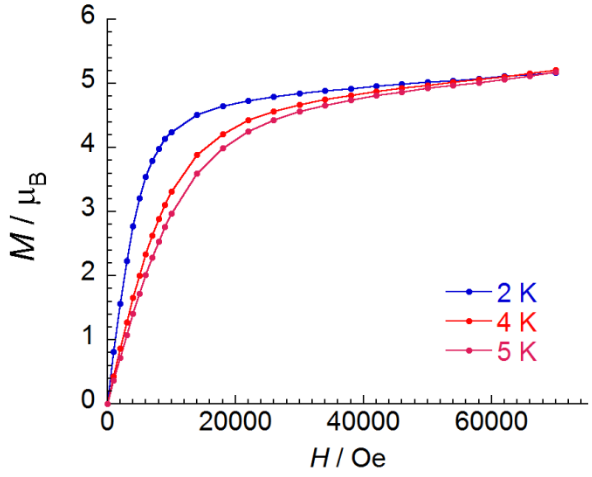

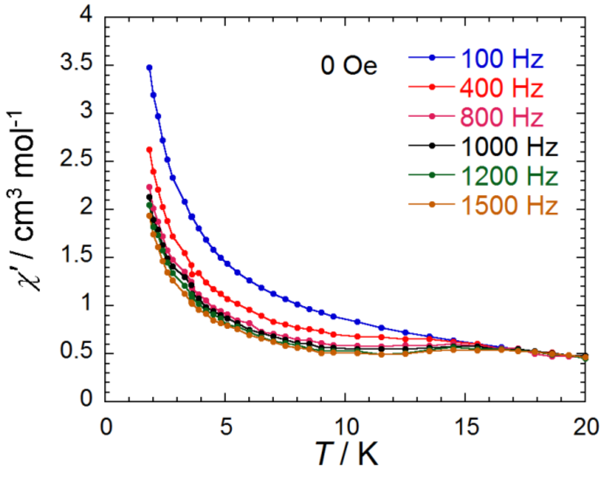

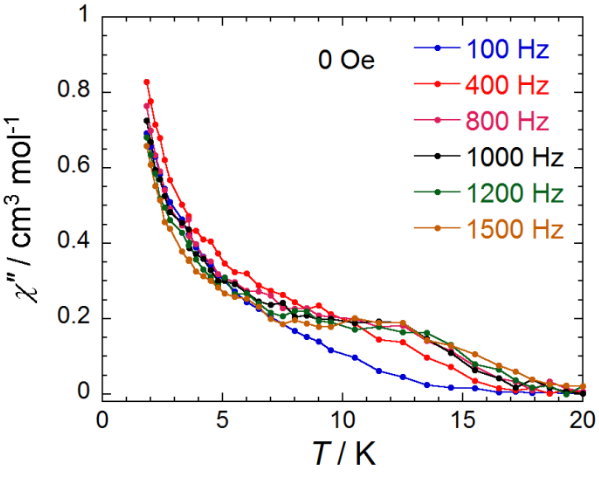

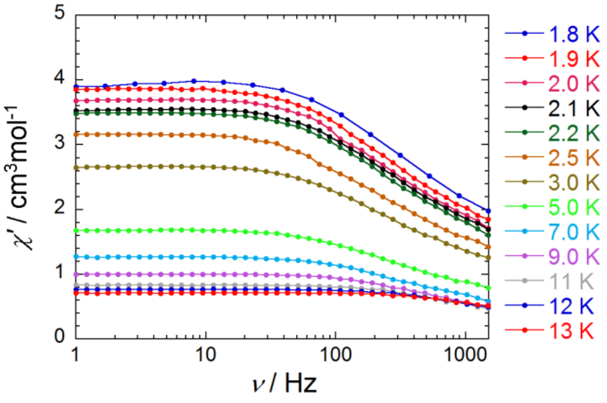

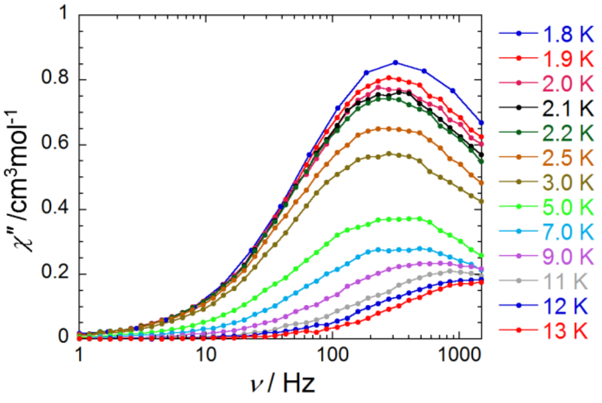

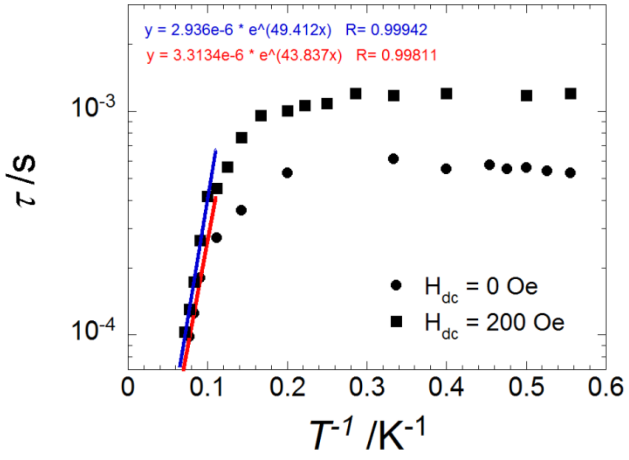
y = 2.936e-6 * e^(49.412x) R= 0.99942
y = 3.3134e-6 * e^(43.837x) R= 0.99811
H_dc = 0 Oe
H_dc = 200 Oe
τ /s
T^-1 /K^-1

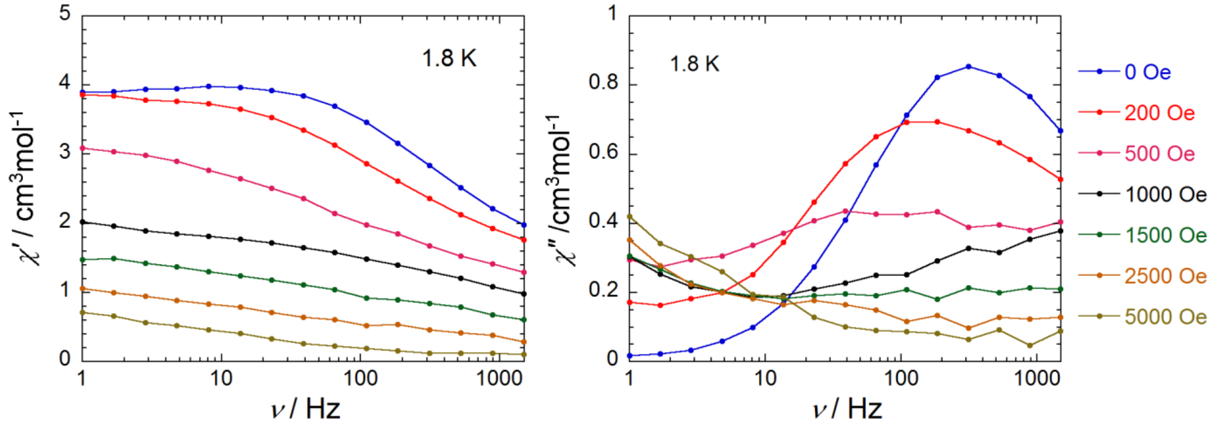
1.8 K
χ' / cm^3mol^-1
ν / Hz
1.8 K
χ" /cm^3mol^-1
ν / Hz
0 Oe
200 Oe
500 Oe
1000 Oe
1500 Oe
2500 Oe
5000 Oe

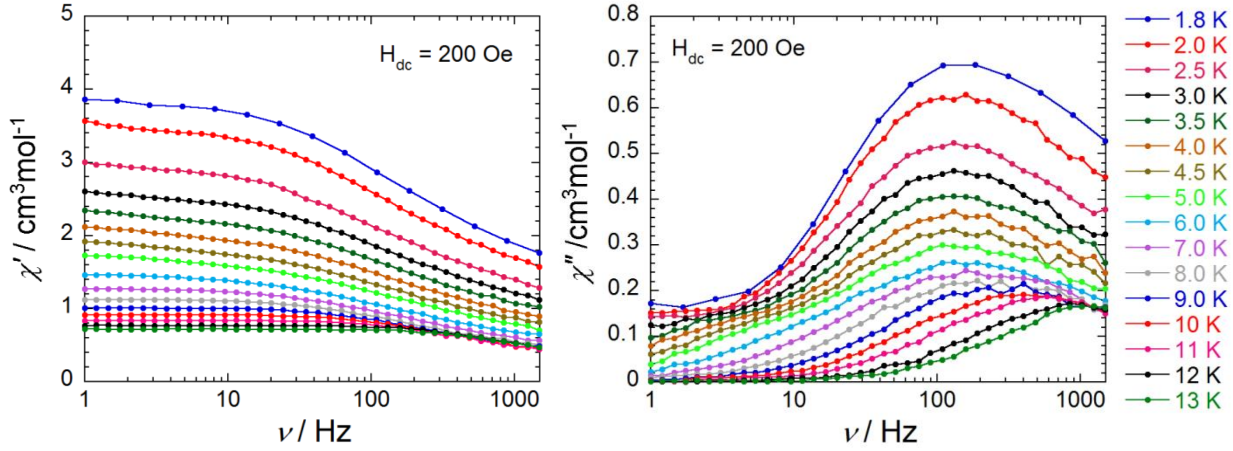
H_dc = 200 Oe
χ' / cm^3mol^-1
ν / Hz
H_dc = 200 Oe
χ" /cm^3mol^-1
ν / Hz
1.8 K
2.0 K
2.5 K
3.0 K
3.5 K
4.0 K
4.5 K
5.0 K
6.0 K
7.0 K
8.0 K
9.0 K
10 K
11 K
12 K
13 K

Lyh13_2C $[DyL_3(HL)]\cdot[DyL_3\cdot EtOH]\cdot 2EtOH$ - compound 2

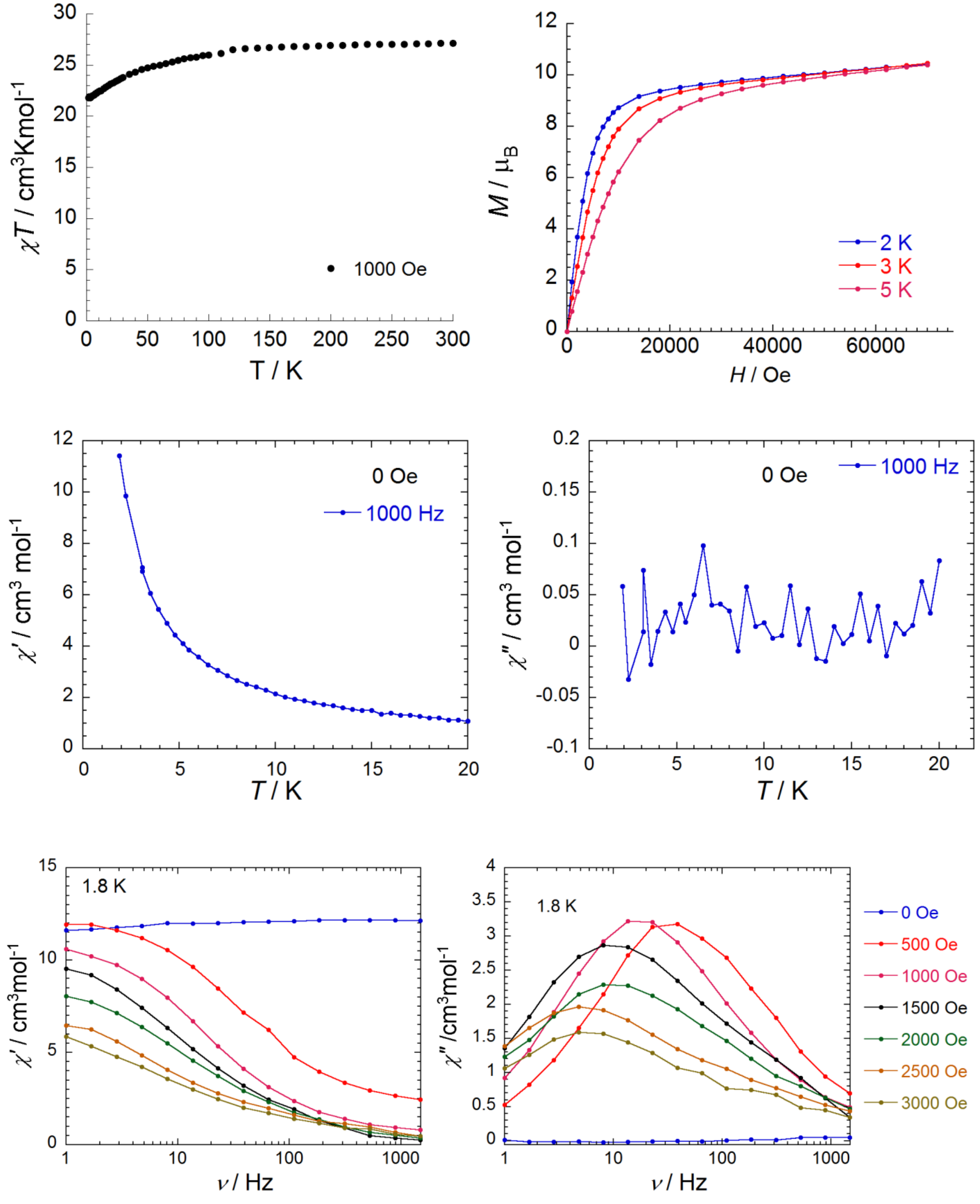

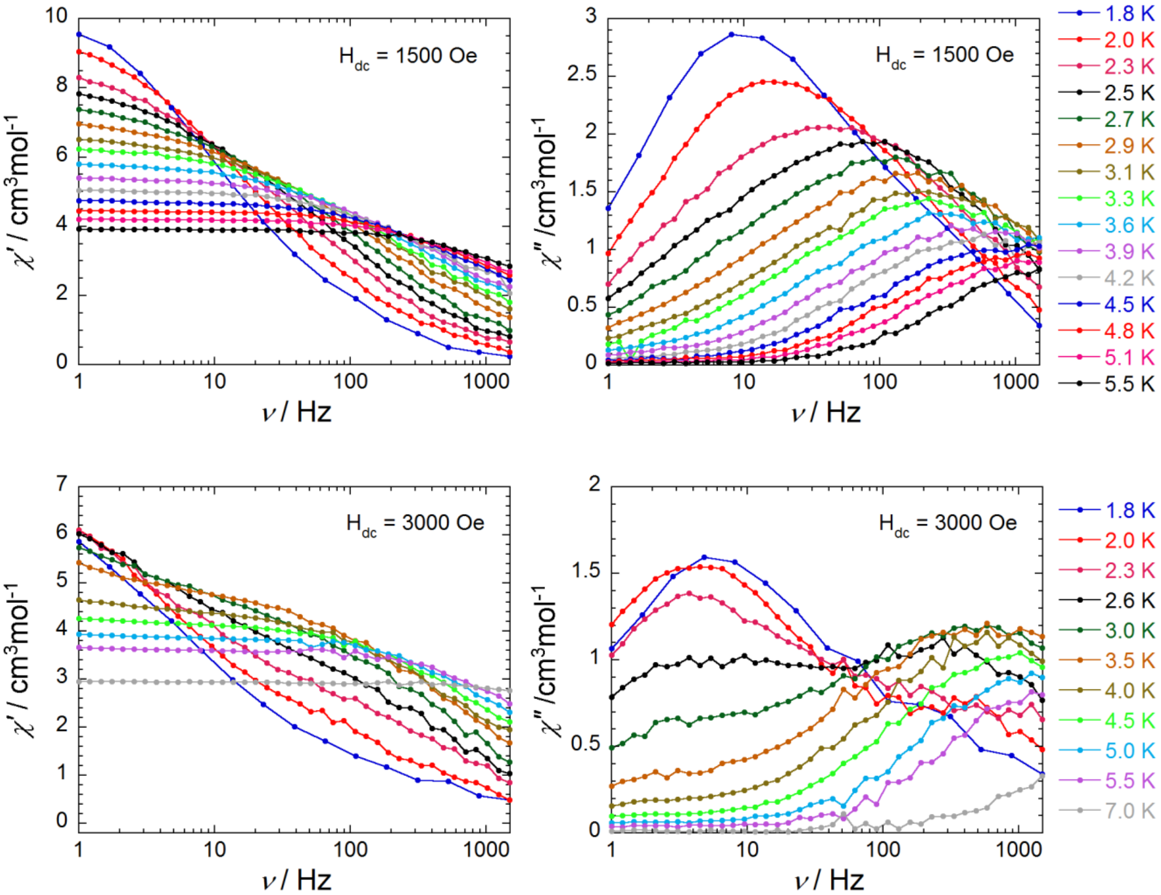
H_dc = 1500 Oe
χ' / cm3mol-1
ν / Hz
χ" /cm3mol-1
1.8 K
2.0 K
2.3 K
2.5 K
2.7 K
2.9 K
3.1 K
3.3 K
3.6 K
3.9 K
4.2 K
4.5 K
4.8 K
5.1 K
5.5 K
H_dc = 3000 Oe
1.8 K
2.0 K
2.3 K
2.6 K
3.0 K
3.5 K
4.0 K
4.5 K
5.0 K
5.5 K
7.0 K

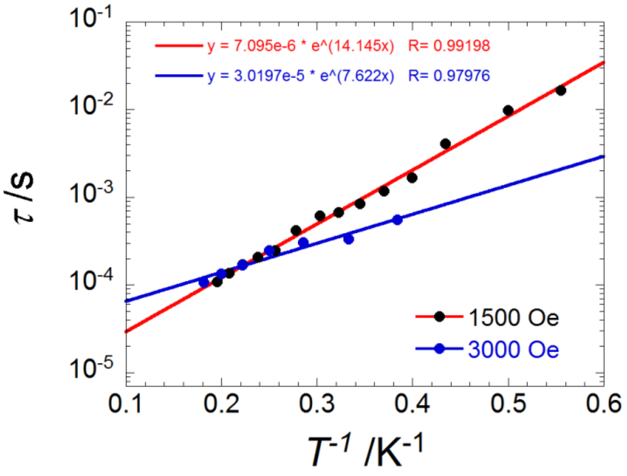
y = 7.095e-6 * e^(14.145x) R= 0.99198
y = 3.0197e-5 * e^(7.622x) R= 0.97976
τ /s
T-1 /K-1
1500 Oe
3000 Oe

LYH41_2 – compound 3

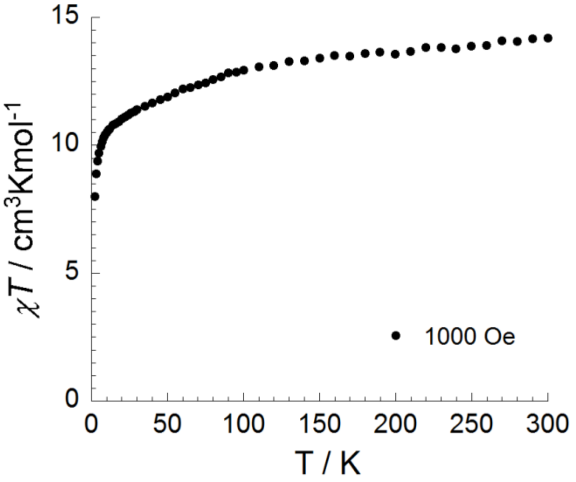

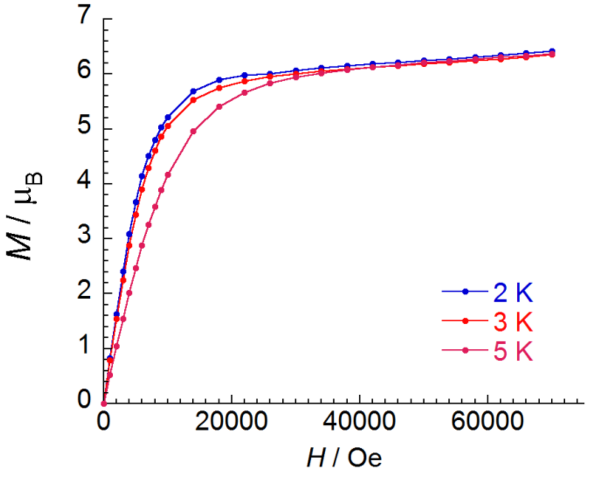

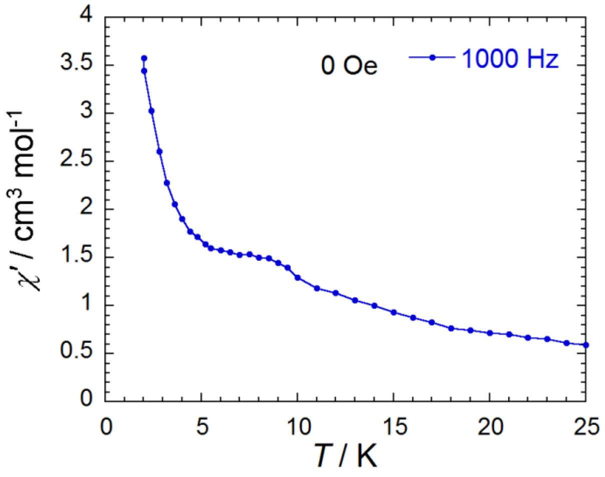

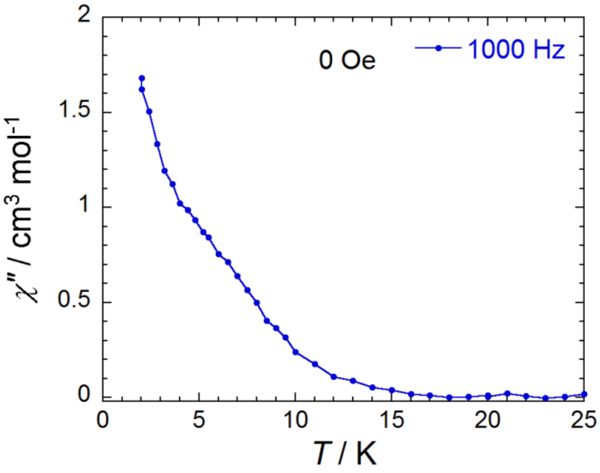

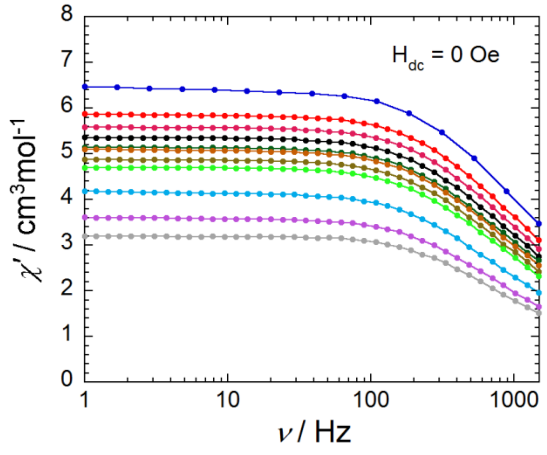

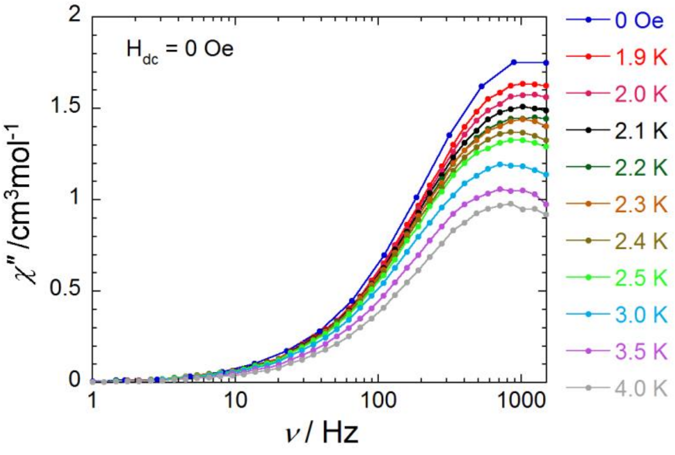

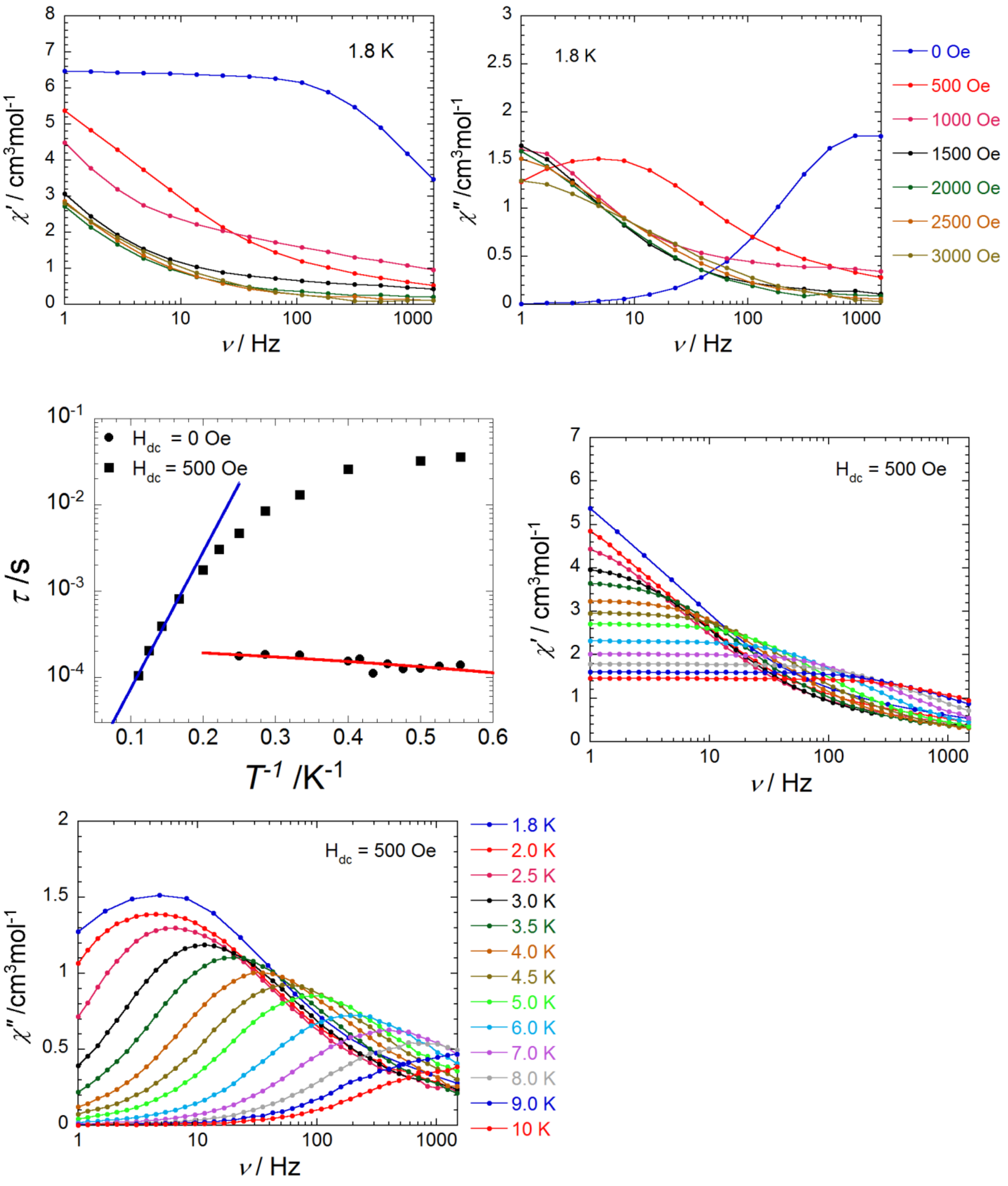
1.8 K
χ' / cm3mol-1
ν / Hz
χ'' /cm3mol-1
0 Oe
500 Oe
1000 Oe
1500 Oe
2000 Oe
2500 Oe
3000 Oe
Hdc = 0 Oe
Hdc = 500 Oe
τ /s
T-1 /K-1
1.8 K
2.0 K
2.5 K
3.0 K
3.5 K
4.0 K
4.5 K
5.0 K
6.0 K
7.0 K
8.0 K
9.0 K
10 K

Table S2: Parameters obtained from fitting of the Cole–Cole diagram (under a DC field of 0 Oe) of compound **1**.

T (K)	α	χ_0 (cm^3/mol)	χ_{inf} (cm^3/mol)	R^2
1.9	0.231(14)	3.892(6)	1.443(39)	0.981
2.0	0.232(21)	3.729(9)	1.355(54)	0.961
2.1	0.189(20)	3.571(7)	1.396(45)	0.963
2.2	0.206(16)	3.516(6)	1.319(36)	0.975
2.5	0.279(15)	3.193(6)	1.067(36)	0.979
3.0	0.196(18)	2.683(5)	1.027(31)	0.968
5.0	0.177(15)	1.690(2)	0.656(16)	0.979
7.0	0.188(14)	1.275(2)	0.477(13)	0.982
9.0	0.160(14)	1.005(1)	0.370(13)	0.985
11	0.136(18)	0.833(0)	0.316(16)	0.981
12	0.147(20)	0.767(0)	0.302(21)	0.974
13	0.073(23)	0.711(0)	0.316(25)	0.960

Table S3: Parameters obtained from fitting of Cole–Cole diagram (under a DC field of 200 Oe) of compound **1**.

T (K)	α	χ_0 (cm^3/mol)	χ_{inf} (cm^3/mol)	R^2
2.0	0.407(12)	3.621(11)	1.109(34)	0.976
2.5	0.444(10)	3.087(9)	0.871(24)	0.983
3.0	0.431(11)	2.676(8)	0.778(23)	0.978
3.5	0.449(11)	2.403(8)	0.665(21)	0.979
4.0	0.470(10)	2.179(7)	0.560(19)	0.981
4.5	0.471(8)	1.965(5)	0.509(15)	0.986

5.0	0.459(6)	1.771(3)	0.477(9)	0.993
6.0	0.400(6)	1.492(2)	0.455(7)	0.995
7.0	0.330(9)	1.286(2)	0.431(8)	0.989
8.0	0.296(14)	1.134(2)	0.387(11)	0.977
9.0	0.239(14)	1.013(2)	0.379(9)	0.982
10	0.213(14)	0.918(1)	0.348(10)	0.982
11	0.177(15)	0.840(0)	0.339(10)	0.983
12	0.111(18)	0.769(0)	0.361(13)	0.976
13	0.125(15)	0.718(0)	0.315(13)	0.986

Table S4: Parameters obtained from fitting of Cole–Cole diagram (under a DC field of 1500 Oe) of compound **2**.

T (K)	α	χ_0 (cm³/mol)	χ_{inf} (cm³/mol)	R^2
2.0	0.426(4)	10.180(36)	0.026 (22)	0.994
2.3	0.458(5)	9.105(34)	0.169(33)	0.989
2.5	0.470(5)	8.471(30)	0.259(40)	0.988
2.7	0.476(5)	7.849(22)	0.279(42)	0.992
2.9	0.462(4)	7.276(13)	0.008(35)	0.996
3.1	0.455(5)	6.751(12)	0.177(43)	0.996
3.3	0.417(9)	6.369(17)	0.623(65)	0.989
3.6	0.402(6)	5.898(8)	0.784(44)	0.996
3.9	0.366(8)	5.461(8)	1.032(52)	0.995
4.2	0.341(15)	5.084(12)	1.146(98)	0.981
4.5	0.317(13)	4.750(8)	1.263(89)	0.988
4.8	0.262(11)	4.456(4)	1.560(60)	0.994

5.1	0.218(10)	4.206(3)	1.691(53)	0.995
5.5	0.179(17)	3.918(3)	1.721(104)	0.982

Table S5: Parameters obtained from fitting of Cole–Cole diagram (under a DC field of 3000 Oe) of compound **2**.

T (K)	α	χ_0 (cm³/mol)	χ_{inf} (cm³/mol)	R^2
3.0				
3.5	0.595(19)	5.586(51)	1.683(425)	0.963
4.0	0.450(19)	4.683(21)	0.105(208)	0.975
4.5	0.368(17)	4.267(11)	0.480(147)	0.983
5.0	0.315(25)	3.927(10)	0.855(200)	0.968
5.5	0.266(34)	3.645(9)	1.042(279)	0.947
7.0				

Table S6: Parameters obtained from fitting of Cole–Cole diagram (under a DC field of 0 Oe) of compound **3**.

T (K)	α	χ_0 (cm³/mol)	χ_{inf} (cm³/mol)	R^2
1.9	0.147(7)	5.86(0)	1.68(6)	0.997
2.0	0.134(7)	5.59(0)	1.64(5)	0.997
2.1	0.136(7)	5.36(0)	1.57(5)	0.997
2.2	0.133(1)	5.15(0)	1.51(5)	0.996
2.3	0.154(7)	5.09(0)	1.37(5)	0.997
2.4	0.135(7)	4.87(0)	1.40(4)	0.997
2.5	0.130(7)	4.70(0)	1.36(4)	0.996
3.0	0.179(8)	4.17(0)	0.94(5)	0.995

3.5	0.153(7)	3.60(0)	0.85(4)	0.995
4.0	0.103(1)	3.20(0)	0.86(3)	0.994

Table S7: Parameters obtained from fitting of Cole–Cole diagram (under a DC field of 500 Oe) of compound **3**.

T (K)	α	χ_0 (cm^3/mol)	χ_{inf} (cm^3/mol)	R^2
2.0	0.468(6)	6.51(5)	0.29(1)	0.996
2.5	0.409(8)	5.34(5)	0.27(1)	0.989
3	0.347(10)	4.37(3)	0.25(2)	0.978
3.5	0.308(9)	3.85(2)	0.23(1)	0.976
4.0	0.276(11)	3.36(1)	0.21(2)	0.971
4.5	0.257(10)	3.04(1)	0.20(2)	0.975
5.0	0.232(11)	2.76(1)	0.22(2)	0.977
6.0	0.199(10)	2.33(0)	0.25(2)	0.987
7.0	0.179(10)	2.03(0)	0.29(2)	0.990
8.0	0.158(10)	1.79(0)	0.36(2)	0.993
9.0	0.117(11)	1.60(0)	0.51(2)	0.991
10	0.130(13)	1.46(0)	0.55(3)	0.988

Determination of the orientation of the magnetic anisotropy with Magellan software

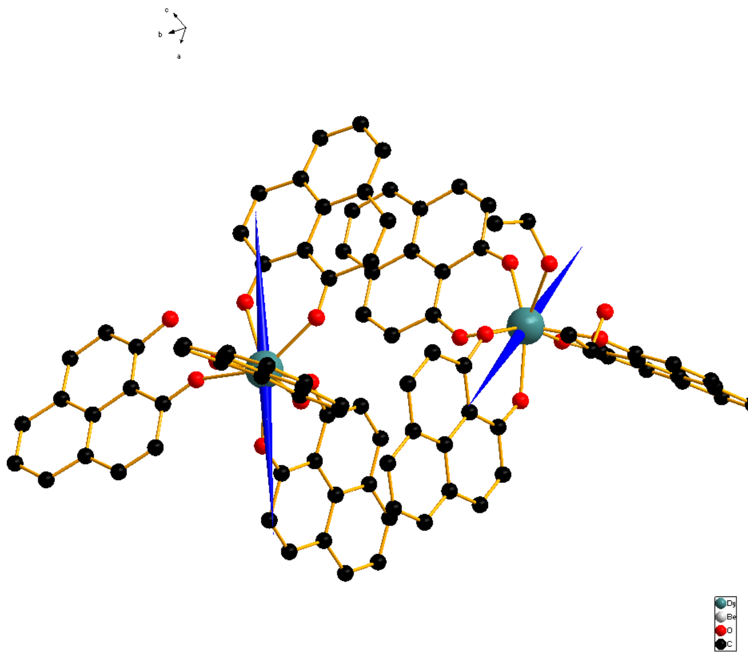

Figure S8: Orientation of the main anisotropy axis in complex **2** indicated as blue arrows calculated using Magellan Software. Coordinates are taken from the crystal structures depicted in Figures 1–3.
